# Pseudopodium-enriched atypical kinase 1 mediates angiogenesis by modulating GATA2-dependent VEGFR2 transcription

**DOI:** 10.1038/s41421-018-0024-3

**Published:** 2018-05-29

**Authors:** Huawei Wang, John Lapek, Ken Fujimura, Jan Strnadel, Bei Liu, David J. Gonzalez, Wei Zhang, Felicia Watson, Vicky Yu, Chao Liu, Carina Muccilo Melo, Yury I. Miller, Kathryn C. Elliott, David A. Cheresh, Richard L. Klemke

**Affiliations:** 1 Department of Pathology, University of California San Diego, La Jolla, CA USA; 2 Moores Cancer Center, University of California San Diego, La Jolla, CA USA; 3 Department of Pharmacology, University of California San Diego, La Jolla, CA USA; 4 Skaggs School of Pharmacy and Pharmaceutical Sciences, University of California San Diego, La Jolla, CA USA; 5 Department of Medicine, University of California San Diego, La Jolla, CA USA; 6 Department of Biochemistry, Universidade Federal de São Paulo, São Paulo/SP, Brazil; 7 Sanford Consortium for Regenerative Medicine, University of California San Diego, La Jolla, CA USA; 8 Present Address: Biomedical Center Martin, Department of Molecular Medicine, Jessenius Faculty of Medicine in Martin, Comenius University in Bratislava, 03601 Martin, Slovakia

## Abstract

PEAK1 is a newly described tyrosine kinase and scaffold protein that transmits integrin-mediated extracellular matrix (ECM) signals to facilitate cell movement and growth. While aberrant expression of PEAK1 has been linked to cancer progression, its normal physiological role in vertebrate biology is not known. Here we provide evidence that PEAK1 plays a central role in orchestrating new vessel formation in vertebrates. Deletion of the PEAK1 gene in zebrafish, mice, and human endothelial cells (ECs) induced severe defects in new blood vessel formation due to deficiencies in EC proliferation, survival, and migration. Gene transcriptional and proteomic analyses of PEAK1-deficient ECs revealed a significant loss of vascular endothelial growth factor receptor 2 (VEGFR2) mRNA and protein expression, as well as downstream signaling to its effectors, ERK, Akt, and Src kinase. PEAK1 regulates VEGFR2 expression by binding to and increasing the protein stability of the transcription factor GATA-binding protein 2 (GATA2), which controls VEGFR2 transcription. Importantly, PEAK1-GATA2-dependent VEGFR2 expression is mediated by EC adhesion to the ECM and is required for breast cancer-induced new vessel formation in mice. Also, elevated expression of PEAK1 and VEGFR2 mRNA are highly correlated in many human cancers including breast cancer. Together, our findings reveal a novel PEAK1-GATA2-VEGFR2 signaling axis that integrates cell adhesion and growth factor cues from the extracellular environment necessary for new vessel formation during vertebrate development and cancer.

## Introduction

New blood vessel formation from the pre-existing vasculature, termed angiogenesis, is essential for proper development in all vertebrates, and its deregulation contributes to many different diseases including cancer^1^. It is now established that the angiogenic process in normal and diseased tissues is primarily driven by VEGFA and its receptor VEGFR2, which transmit signaling cascades that direct endothelial cell (EC) spouting, migration, and proliferation, as well as cytoskeletal changes needed for proper tube formation^2^. In addition to VEGFA-VEGFR2 signaling, new blood vessel construction requires ECs to adhere to, remodel, and invade through dense arrays of complex extracellular matrix (ECM) proteins present in the basement membrane and tissue parenchyma^3^. ECM proteins are particularly important in angiogenesis because they transmit crucial positional and mechanical signals to the interior of the cell which orchestrate EC movement, growth, differentiation, and survival^3^. Thus, it is crucial that ECs can sense and respond to cues from both ECM proteins as well as growth factors present in the extracellular environment. In fact, pharmacological and therapeutic targeting of VEGFR2 or ECM-mediated focal adhesion signaling potently inhibits vascular formation in vitro and blocks angiogenesis in preclinical animal models^3–5^. Therefore, it is important to understand in detail how ECM proteins and VEGFR2 signaling coordinately regulate EC biology under normal and diseased states.

Pseudopodium-enriched atypical tyrosine kinase 1 (PEAK1, Sgk269) is a newly discovered non-receptor tyrosine kinase ubiquitously expressed in all tissues, highly conserved in vertebrates, and is deregulated in many different cancers^6^. PEAK1 regulates focal adhesion dynamics and integrin signaling to the actin cytoskeleton in breast and pancreatic cancer cells^7^. Biochemically, PEAK1 interacts with Src, Shc1, Crk, and Grb2 adaptor proteins in the cytoplasm to convey signals from membrane receptor tyrosine kinases such as EGFR and ErbB2, which mediate cancer cell migration and proliferation^7–10^. Recently, PEAK1 was shown to interact with and regulate YAP1/TAZ in pancreatic cancer cells^11^. YAP1/TAZ is a central cytoskeletal tension sensor and transcriptional co-activator that controls cell proliferation, shape, and size in response to biomechanical signals from the ECM^12^. These accumulating data indicate that PEAK1 serves as a scaffold to integrate and transmit multiple signals from the extracellular environment to the interior of cancer cells. However, to fully understand PEAK1′s role in cancer it is necessary to understand its normal physiological role in the body, which is not yet known. Here we investigate the normal physiological function of PEAK1 in PEAK1 knockout mice, zebrafish, and human ECs. PEAK1 was found to regulate vertebrate angiogenesis by modulating GATA2-mediated VEGFR2 transcription and downstream signaling in response to ECM signaling. In addition, interrogation of cancer databases revealed that elevated levels of PEAK1 and VEGFR2 mRNA are highly correlated in many human cancers, suggesting that the regulation of the PEAK1-VEGFR2 axis contributes to this disease.

## Results

### PEAK1 is highly conserved in vertebrates and is important for angiogenesis

Zebrafish is a well-established model organism to study vertebrate development, genetics, and angiogenesis. Zebrafish embryos are transparent, develop rapidly ex vivo, and are readily amenable to genetic manipulation and microscopy imaging, and thus are an ideal model to study the physiological role of PEAK1 in vertebrates. The zebrafish *peak1* gene (Gene ID: 565111) encodes a protein with 1695 amino acids (aa), which is slightly shorter than the proteins encoded by the mouse *Peak1* gene (Gene ID: 244895, 1735 aa) and the human *PEAK1* gene (Gene ID: 79834, 1746 aa). The percentage of sequence identity of PEAK1 proteins of *Homo sapiens*, *Mus musculus*, and *Danio rerio* is 44.5%, while the percentage of consensus positions is 92.0% (Supplementary Fig. S1a). Moreover, PEAK1′s protein interaction domains and phosphorylation sites are highly conserved in vertebrates, which include the actin binding domain (aa 339–727), the putative tyrosine kinase domain (aa 1330–1664), and the Src family kinase consensus phosphorylation sites (aa Y635 and Y665) (Supplementary Fig. S1b). Thus, PEAK1 is well conserved among vertebrates and likely to play an important role in vertebrate physiology.

**Fig. 1:**
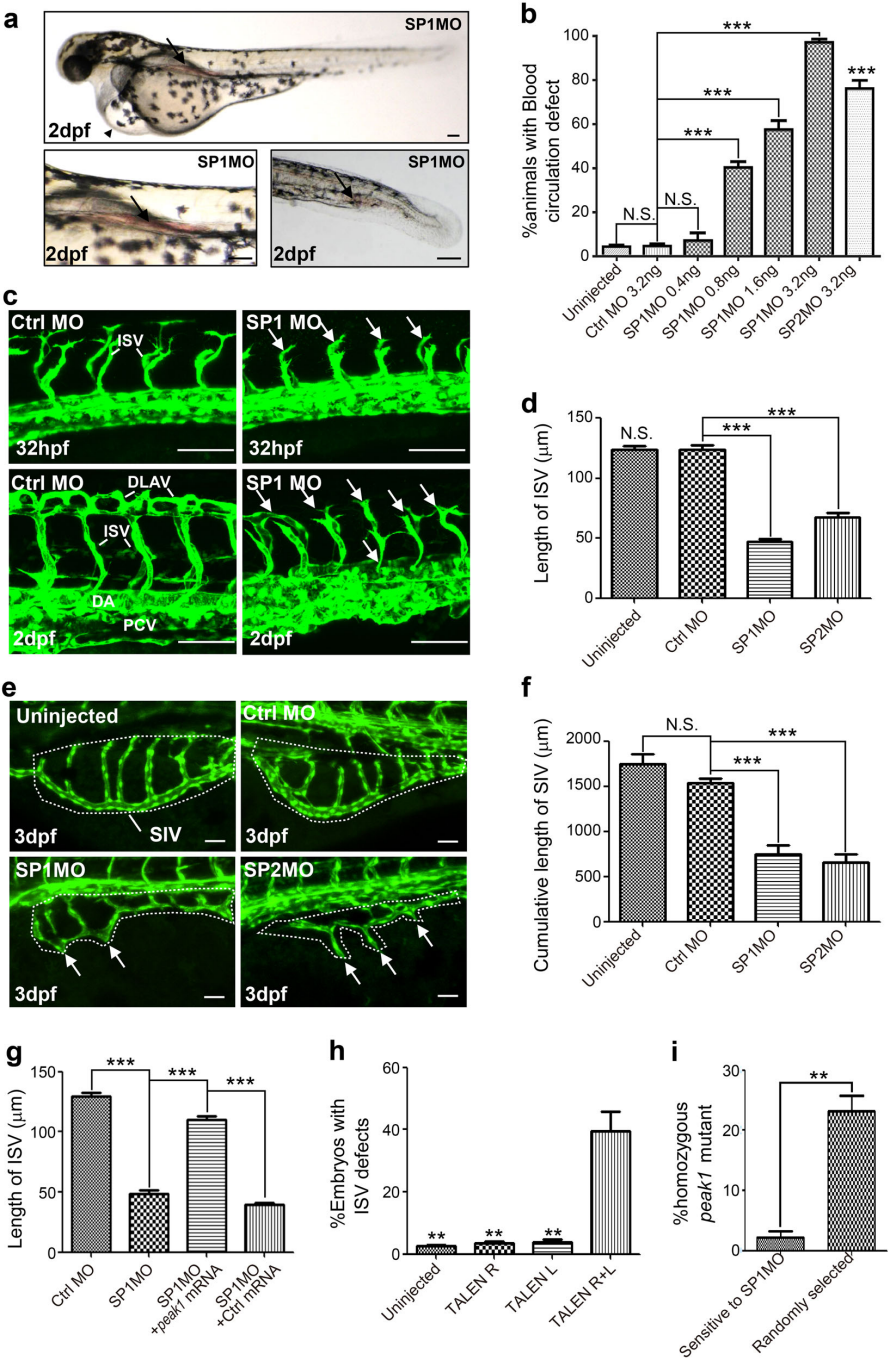
*peak1* is required for vascular development in zebrafish embryos. **a** Bright-field images of pericardial edema (upper panel, arrowhead) and blood accumulation in the anterior aorta as well as in the tail region (arrows) of *peak1* morpholino (MO) knockdown embryos (SP1MO). Pictures were taken at 2 days-post-fertilization (2dpf). Also see Supplementary Fig.S2c. **b** Bar graph shows the percentage of animals with vascular defects caused by control (Ctrl MO) or two different *peak1* specific morpholinos SP1MO and SP2MO. Mean ± SEM; *n* = 30. **c** Confocal images at indicated time points of vasculature formation of *Tg(fli1:egfp)*^*y1*^ zebrafish embryos treated as in (**a**). ISVs = intersegmental vessels. DLAVs = dorsal longitudinal anastomotic vessels. DA = dorsal aorta. PCV = posterior cardinal vein. hpf = hours-post-fertilization. Arrows point to disorganized endothelial sprouts and vessel structures. **d** Bar graph depicts the average ISV length at 32 hpf of MO-treated embryos. Mean ± SEM; *n* = 50. **e** Confocal images of subintestinal vessels (SIVs, dashed line circled region) were captured at 3dpf from embryos treated as in (**c**). Arrows show SIV defects. **f** Bar graph shows the cumulative length of SIVs per embryo from embryos treated as in (**e**); Mean ± SEM; *n* = 10. **g** Bar graph represents the average ISV length at 32hpf of embryos treated as in (**c**) and co-treated with *peak1* mRNA or a nonspecific control mRNA (Ctrl mRNA). Mean ± SEM; *n* = 50. **h** Bar graph shows the percentage of *Tg(fli1:egfp)*^*y1*^embryos with mosaic disrupted ISV defects after treatment of *peak1* specific transcription activator-like effector nuclease (TALEN) mRNAs. TALEN R = right arm (120 pg); TALEN L = left arm (120 pg); TALEN R + L = both arms (60 pg each); Mean ± SEM; *n* = 90. **i** Embryos from incrossed heterozygous *peak1* ^*∆2/ +*^ zebrafish were injected with SP1MO at one-cell stage, subsequently selected at 72 hpf for the vascular defects (Sensitive to SP1MO) or randomly selected and all genotyped individually. Bar graph represents percentage of homozygous *peak1*^*∆2/ ∆2*^ in each group. Mean ± SEM; *n* = 90. All the data are representative of at least three independent experiments. ***, *P* < 0.001; **, *P* < 0.01; N.S., not significant; in (**b**, **d**), vs. Ctrl MO; in (**h**), vs. TALEN R + L. Scale bar = 50 μm

To determine the role of *peak1* in early zebrafish development, we used two different splice-blocking morpholinos (MOs, SP1MO, and SP2MO) to knockdown *peak1* expression in fertilized embryos (Supplementary Fig. S2a). The knockdown efficiency of MOs was confirmed by quantitative real-time RT-PCR (qPCR) and western blotting (WB) (Supplementary Fig. S2d and e). The *peak1* knockdown embryos developed normally for the first 24 h-post-fertilization (hpf) and displayed normal gastrulation, somite, and body formation. However, by 2 days-post-fertilization (dpf) *peak1*-depleted deficient embryos showed prominent vascular defects characterized by pericardial edema and blood accumulation in the anterior aorta and tail region (Fig. 1a, b). These animals survived for 10–12 dpf before succumbing to vascular problems. The primitive brain, notochord, somites, and overall body pattern were grossly normal during early development. These findings indicate that peak1 may play a role in vasculature development, but is largely dispensable for other morphological development.

To further characterize the observed vascular defects in peak1-knockdown embryos, we utilized *Tg(fli1:egfp)*^*y1*^ transgenic zebrafish, which express GFP throughout their vascular system making them readily amenable to high resolution intravital confocal microscopy^13^. This animal model has been widely used to characterize vessel formation and dissect the genetic programs that regulate vertebrate angiogenesis. Interestingly, imaging of the developing vasculature in *peak1*-depleted embryos at 32 hpf revealed major defects in extension of intersegmental vessels (ISV) from the dorsal aorta (DA) or posterior cardinal vein (PCV) (Fig. 1c, d). Endothelial tip cells and stalks initially sprouted from DA or PCV, but showed stunted extension with numerous disorganized cellular projections, which failed to connect to the dorsal longitudinal anastomotic vessels (DLAV). In addition, the subintestinal vessels (SIV) did not extend properly from the duct of Cuvier, preventing vascular plexus formation in the abdominal region (Fig.1e, f). Importantly, injection of *peak1* mRNA into developing embryos rescued the ISV vascular defects induced by SP1MO, reducing the possibility of off-target effects (Fig. 1g). These findings indicate that *peak1* is necessary for proper vascular development in zebrafish embryos.

To independently confirm the *peak1* morpholino-induced vascular defects, we designed a pair of Transcription Activator-like Effector Nucleases (TALEN) for *peak1* gene using a TALEN scaffold toolbox optimized for zebrafish^14^. The designed targeting site locates at exon 1 of the zebrafish *peak1* gene (Supplementary Fig. S2b). TALEN mRNAs were injected into wildtype embryos at the one-cell stage and individual F0 founder embryos were genotyped at 2dpf. The genotyping results suggest the somatic targeting efficiency ranges from ~10 to ~60% (Supplementary Fig. S2f). Previous studies suggest high targeting efficiency in F0 founder embryos can result in biallelic gene targeting in some somatic cells^15^. Interestingly, we did observe that ~ 30% of *peak1* TALEN-injected embryos showed similar blood circulation defects as the *peak1* morphants (Supplementary Fig. S2g and h). Next, we injected *peak1* TALEN mRNAs into *Tg(fli1:egfp)*^*y1*^ embryos. Notably, while about 40% of the embryos injected with TALEN mRNAs targeting both arms (TALEN R + L) showed mosaic ISV defects, injection of same amount of single arm TALEN mRNA (TALEN R or TALEN L) had no effect (Fig. 1h and Supplementary Fig. S2i). These results suggest the mosaic vascular defects caused by *peak1* TALEN injection may be due to genetic ablation of zebrafish *peak1* gene in the somatic cells.

We next outcrossed the F0 founder fish of *peak1* TALEN to generate F1 progeny. Sequencing and genotyping of these animals revealed various point and frameshift mutations in the *peak1* gene (Supplementary Fig. S2j). We focused on the F1 line #2–6, which has a 2 base pair frameshift in *peak1* coding region. The line # 2–6 zebrafish were outcrossed for two generations, followed by backcrossing to obtain homozygous mutants (*peak1*^*∆2/ ∆2*^). Interestingly, although homozygous *peak1*^*∆2/ ∆2*^ is a null mutant as embryos do not express peak1 protein (Supplementary Fig. S2k), these animals do not display vascular defects and survive to become fertile adults (data not shown). The discrepancy in the phenotypic differences between TALEN-induced genetic peak1 mutations and splicing-MO-induced peak1 depletion have two possible sources. First, the vasculature defects induced by the peak1 MO could be due to off-target effects, but this was considered unlikely because we obtained similar phenotypes with two independent MOs, and the addition of *peak1* mRNA rescued the vascular defects in these animals. Second, it is possible that genetic compensation occurred subsequent to the introduction of deleterious peak1 mutations induced by TALENs, and such compensations are not uncommon in genetic deleterious animal models^16^. To investigate possible compensation due to deleterious peak1 null mutations, we used a similar strategy described by Rossi et al.^16^. We reasoned that if the peak1 MO specifically targeted zebrafish *peak1* gene and genetic compensation occurred in *peak1* null mutants (*peak1*^*∆2/ ∆2*^), the peak1 MO should not cause vascular defects in *peak1* null mutant. To test this hypothesis, the progeny from incrosses of *peak1*^*∆2/+*^ heterozygotes were injected with SP1 MO at the one-cell stage. At 72 hpf, two groups of embryos were separated and individually genotyped. Group I included only embryos exhibiting vascular defects (i.e., ISV defects, reduced circulation loop and/or pericardial edema, means embryos are sensitive to peak1 MO), whereas Group II embryos were randomly selected (control group with theoretical Mendelian distribution of genotypes). Based on genotyping, there were much fewer *peak1*^*∆2/ ∆2*^ null mutants in group I than the control group II, suggesting *peak1*^*∆2/ ∆2*^ mutants are indeed much less sensitive to the peak1 MO (Fig. 1i). According to the newly published “Guidelines for morpholino use in zebrafish”^17^, we conclude that peak1 MOs specifically target zebrafish *peak1* gene and homozygous *peak1*^*∆2/ ∆2*^ zebrafish are viable and lack vascular defects due to genetic compensation. Taken together, our results indicate *peak1* is required for proper vascular formation in zebrafish embryos.

### *peak1* cell-autonomously regulates vegfa-induced EC migration and proliferation during vessel formation in zebrafish

**Fig. 2:**
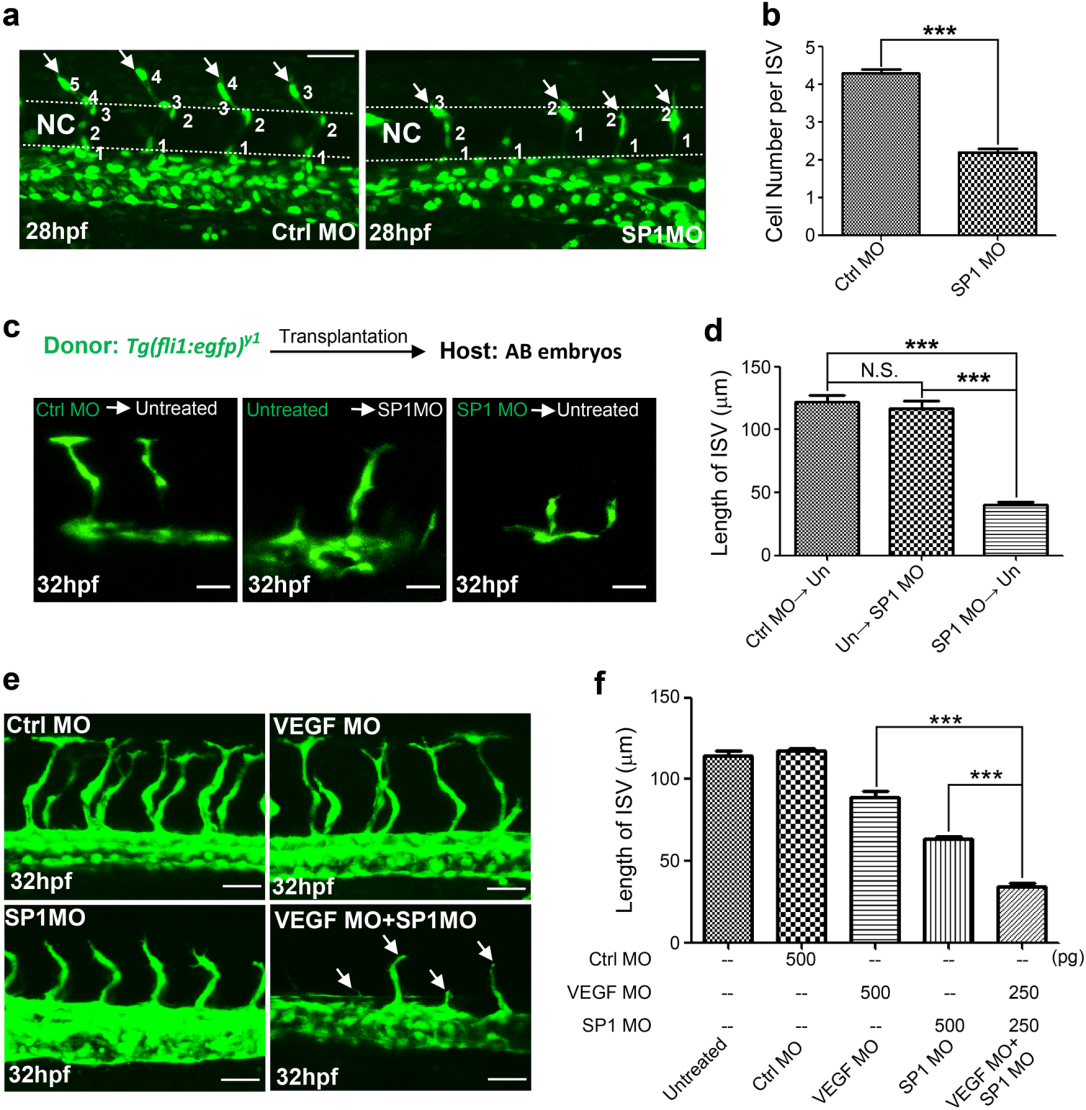
*peak1* is required for endothelial cell (EC) proliferation and migration in zebrafish early development. **a** Confocal images of labeled ISV ECs at 28hpf of *Tg(fli:nls-egfp)* embryos treated with MOs. Also see Video S1 and S2. Arrows point to tip cells sprouting from DA. Dashed line = notochord (NC). **b** Bar graph represents the average number of ECs per ISV. Mean ± SEM; *n* = 30. **c** Endothelial precursor cells were isolated from sphere stage *Tg(fli1:egfp)*^*y1*^ embryos (donor) and transplanted into same stage wild type AB host embryos (host). Either donor or host embryos were treated with indicated MOs at one-cell stage. ISV formation from engrafted GFP ECs were imaged using confocal microscopy at 32hpf. **d** Bar graph shows the average length of GFP labeled ISVs from embryos treated as in (**c**). Mean ± SEM; *n* = 10. All data are representative of at least three independent experiments. **e** Confocal images of ISVs of *Tg(fli1:egfp)*^*y1*^ embryos treated with sup-optimal amount of Ctrl MO, SP1MO, VEGF MO or indicated combination. Arrows show stunted ISVs. **f** Bar graph shows the average length of ISVs from embryos treated as in (**e**). Mean ± SEM; *n* = 30. ***, *P* < 0.001; N.S., not significant. Scale bar = 30 μm

We next investigated how *peak1* regulates zebrafish ISV formation in vivo. Previous work using time-lapse imaging of vessel development in zebrafish embryos shows that ISV formation requires cell sprouting from DA or PCV, tip cell migration, and cell proliferation of a defined number of ECs^13^. To determine how *peak1* depletion impacts these different processes, we performed time-lapse intravital imaging of transgenic *Tg(fli1:nls-egfp*) zebrafish embryos that express GFP in the nucleus of all ECs with or without *peak1* depletion. Interestingly, while endothelial tip cells can sprout from the DA in *peak1*-deficient animals, they displayed severely impaired migration around the notochord compared to control animals (Fig. 2a, Supplementary Movie S1 and S2). Furthermore, *peak1*-depleted animals showed a significant reduction of the total number of ECs per each ISV compared to controls (Fig. 2a, b). These findings suggest that *peak1* controls EC migration and proliferation during zebrafish ISV formation in vivo.

To determine if *peak1* functions cell-autonomously in the ECs during ISV formation, cells from *Tg*(*fli1:egfp*)^y1^ embryos were isolated and transplanted into recipient wild type host embryos at early sphere stage (see schematic Supplementary Fig. S3). Donor cells from control animals injected into host embryos with *peak1* depletion formed normal ISVs, whereas *peak1*-depleted donor cells injected into control embryos formed disorganized and stunted ISVs (Fig. 2c, d). Altogether, these findings indicate that *peak1* regulates EC migration and proliferation during zebrafish ISV development in a cell-autonomous manner.

The vascular defects observed in the *peak1*-depleted zebrafish embryos were similar to the cardiac edema and blood pooling defects described in zebrafish models of perturbed vegfa/vegfr2 signaling^18^. Importantly, vegfa/vegfr2 signaling plays a prominent role in the development of SIV and ISV vessels in zebrafish^5^. Therefore, we investigated a possible role for peak1 in regulating vegfa/vegfr2 signaling and angiogenesis. *Tg*(*fli1:egfp*)^y1^ embryos were co-injected with suboptimal doses of MOs to *peak1* and/or *vegfa* and then monitored for vessel formation. Animals injected with suboptimal doses of either *peak1* or *vegfa* MO alone showed only minor vessel defects, whereas animals co-injected with both MOs showed dramatic loss of ISVs (Fig. 2e, f). These findings suggest that *peak1* and *vegfa* may act synergistically to mediate ISV formation.

### PEAK1 regulates VEGFA-induced human EC migration, proliferation, and survival as well as vessel morphogenesis in vitro

**Fig. 3:**
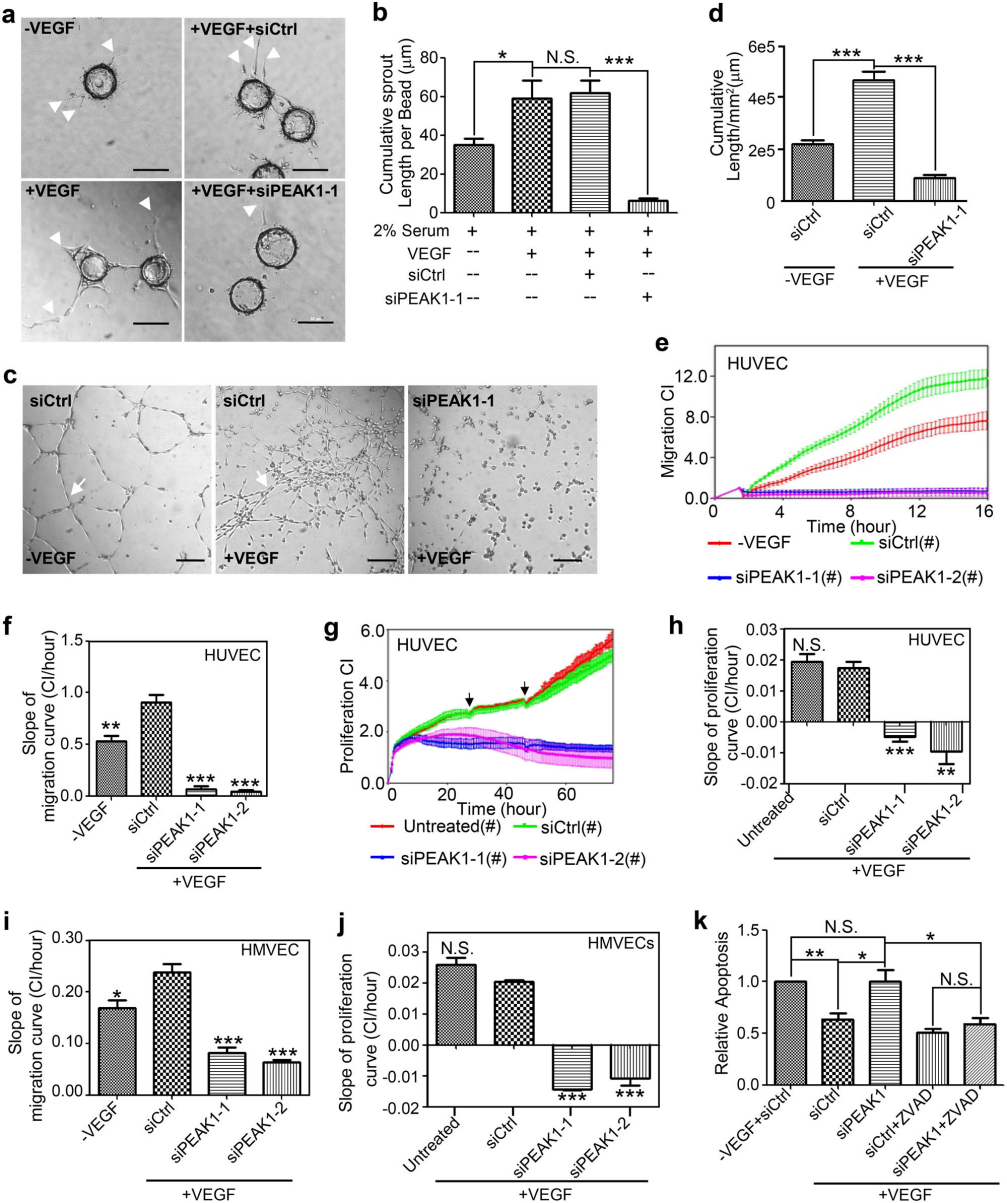
PEAK1 is required for Vascular Endothelial Growth Factor A (VEGFA)-induced proliferation, migration and morphogenesis of human ECs in vitro. **a** Phase-contrast images of fibrin gel sprouting assay with Human Umbilical Vein Endothelial Cells (HUVECs) transfected with control (siCtrl) or PEAK1 (siPEAK1-1) siRNA and treated with (+VEGF) or without VEGF (-VEGF). Arrowheads show vessel sprouts radiating from the bead surface. **b** Bar graph represents the cumulative length of sprouts per bead. Mean ± SEM; *n* = 10. **c** Phase-contrast images of Matrigel tube-forming assay with HUVECs treated with siRNAs in the presence or absence of VEGF. Arrows show vascular tubes. (**d**) Bar graph represents cumulative length of vascular tubes (per mm^2^) formed as in (**c**). Mean ± SEM; *n* = 5. Real-time cell migration (**e**, **f**, **i**) or proliferation (**g**, **h**, **j**) kinetics were measured by xCELLigence electrical impedance system of HUVECs (**e**–**h**) or Human Cardiac Microvascular Endothelial Cells (HMVECs, **i**,** j**) treated with indicated siRNAs. The chambers were coated with collagen I. Bar graph represents the slopes of the migration curves (**f**, **i**, from 2 to 12 h) or proliferation curves (**h**, **j**, from 4 to 72 h) of HUVECs and HMVECs. CI = Cell Index; Mean ± SEM of quadruplicate wells. (**k**) HUVECs were transfected with indicated siRNA, treated with or without VEGF and co-treated with or without zVAD, a pan-caspase inhibitor. Apoptosis was then measured by FACS with staining of 7-AAD and FITC-Annexin-V. Bar graph represents relative apoptotic cell ratio normalized with (–VEGF + siCtrl) group. Mean ± SEM; *n* = 3. All data are representative of at least three independent experiments. ***P < 0.001; **P < 0.01; *P < 0.05; N.S., not significant; (**f**, **h**, **i**,** j**), vs. siCtrl + VEGF group. Scale bar = 10 μm

To further explore the potential role of PEAK1 in VEGFA-mediated EC functions, we first used a well-established 3D bead sprouting angiogenesis assay with primary human umbilical vein endothelial cells (HUVECs)^19^. This model provides a defined ECM microenvironment and growth factor stimuli for the ECs, and recapitulates all the key stages of angiogenesis including EC sprouting, ECM invasion, migration, proliferation, lumen formation, and microcapillary branching and anastomosis. HUVECs were depleted of PEAK1 by siRNA treatment (Supplementary Fig. S4a) and allowed to attach to Cytodex beads, which were then imbedded in fibrin gels and overlaid with fibroblasts. Cell cultures were then treated with or without recombinant human VEGFA (VEGF-165) to induce sprouting and vessel formation. PEAK1-depleted HUVECs showed significantly reduced VEGFA-induced vessel sprouting compared to control cells (Fig. 3a, b). In addition, PEAK1 depletion inhibited VEGFA-induced tube formation in a standard EC tube-forming assay using Matrigel-coated dishes (Fig. 3c, d). These results suggest PEAK1 cell-autonomously regulates VEGFA-induced morphogenesis of ECs in vitro.

To determine how PEAK1 facilitates vessel formation in vitro, we monitored the real-time EC migration and proliferation with the xCELLigence biosensor technology under PEAK1-depleted conditions. The results suggest PEAK1 depletion strongly inhibited VEGFA-induced cell migration and proliferation in both HUVECs and primary human cardiac microvascular endothelial cells (HMEVCs) (Fig. 3e–j and Supplementary Fig. S4c and d). Consistent with these findings, peak1 knockdown in *Tg(fli1:nls-egfp*) zebrafish embryos inhibited EC migration and proliferation in vivo (Fig. 2a, b). Importantly, the inability of PEAK1-depleted cells to migrate and proliferate in vitro was not due to their inability to attach to the collagen-coated plates (Supplementary Fig. S4b). In addition, VEGFA-induced cell survival was significantly inhibited in PEAK1-depleted HUVECs (Fig. 3k). Importantly, treatment with cell permeable zVAD-FMK, a pan caspase inhibitor, rescued the apoptotic response in PEAK1-depleted HUVECs, indicating that cell death was due to caspase-mediated apoptosis (Fig. 3k). Altogether, these results indicate that PEAK1 regulates VEGFA-induced EC vessel morphogenesis in vitro by mediating EC growth, survival and migration.

### PEAK1 regulates VEGFR2 protein expression as well as downstream signaling in human ECs

**Fig. 4:**
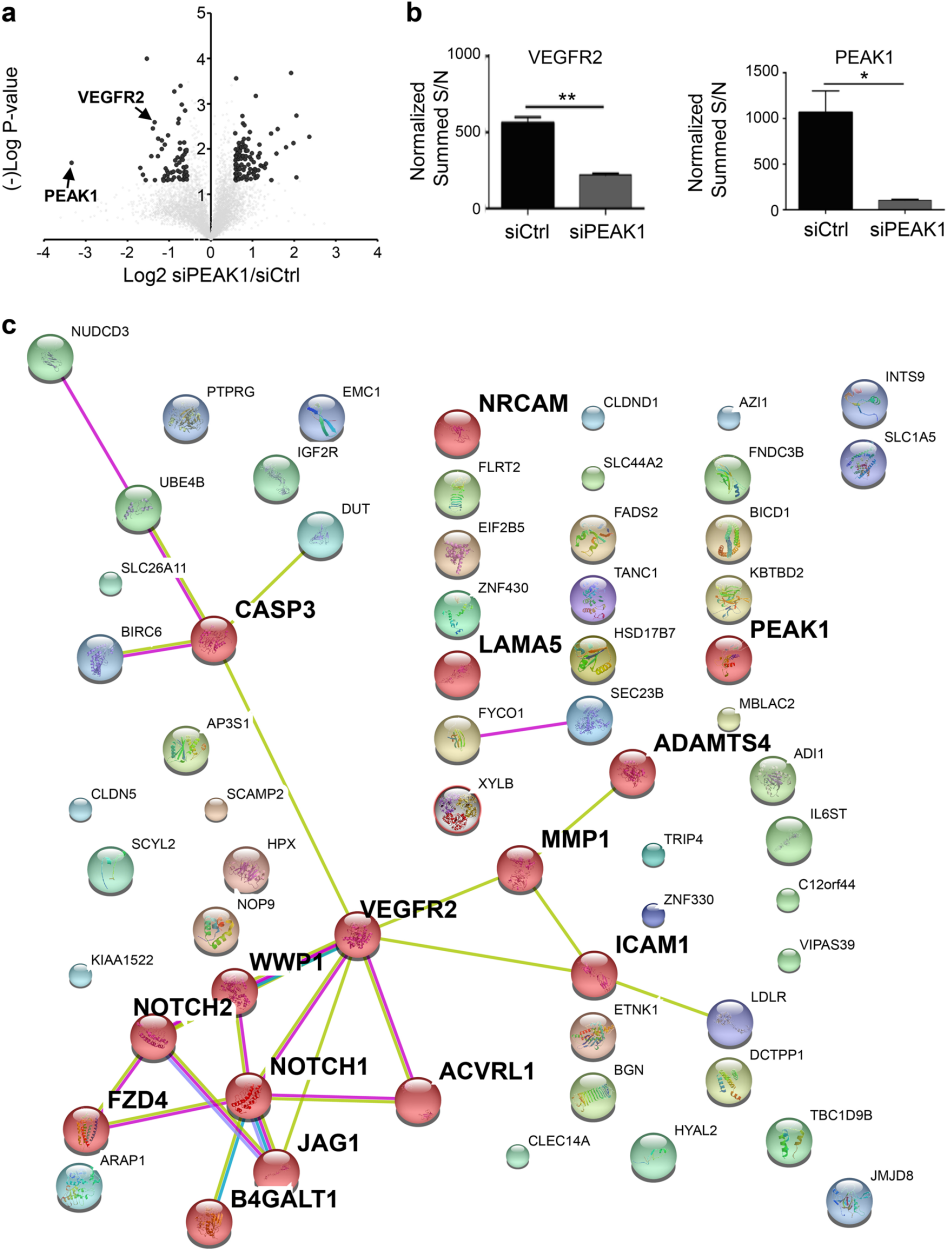
PEAK1 regulates the expression of multiple pro-angiogenic factors in human ECs. **a** Volcano plot for quantitative multiplexed proteomics revealed 5565 proteins across all HUVECs samples treated with siCtrl or siPEAK1 (Also see Supplementary Table S4). A total of 103 proteins were significantly up-regulated while 63 proteins were significantly down-regulated in the siPEAK1 samples relative to the siCtrl samples, (*n* = 3, *p* < 0.05, fold-change ≥1.5, dark dots, also see Supplementary Table S5). **b** Bar graph represents the normalized summed S/N value of VEGFR2 (Left) or PEAK1 (Right) protein level identified by multiplexed proteomics. Mean ± SEM; *n* = 3; **P < 0.003; *P < 0.02. **c** Protein network analysis using STRING10.0 of downregulated proteins in HUVECs by *PEAK1* depletion from quantitative multiplex proteomic analysis. Proteins with known or predicted functions in vascular development or angiogenesis are highlighted (Red ball)

To gain a mechanistic understanding of how PEAK1 alters EC functions, we profiled the protein networks altered in PEAK1-depleted HUVECs. For these studies, we used quantitative multiplexed proteomics and isobaric labeling with tandem mass tags (TMT)^20^. This technology facilitated simultaneous and quantitative analyses of three biological replicates from both PEAK1-depleted and mock treated control HUVECs. Only proteins with more than 1.5-fold differences in the control samples relative to the PEAK1-depleted samples with a *P* value < 0.05 were considered significantly changed by PEAK1-depletion. A total of 5565 proteins were identified across all groups (Fig. 4a; Supplementary Table S4). PEAK1-depleted HUVECs showed 63 down-regulated and 103 up-regulated proteins (Supplementary Table S5). The functional relevance of proteins altered by PEAK1 loss was classified using Gene Ontology (GO) and network analysis. Of the 63 down-regulated proteins, 13 proteins have known or predicted functions in vasculature development or angiogenesis, and the majority are proangiogenic proteins including Jagged1 (JAG1), Matrix metalloproteinase-1 (MMP1), Intercellular Adhesion Molecule 1 (ICAM1) and VEGFR2 (Fig. 4b, c).

**Fig. 5:**
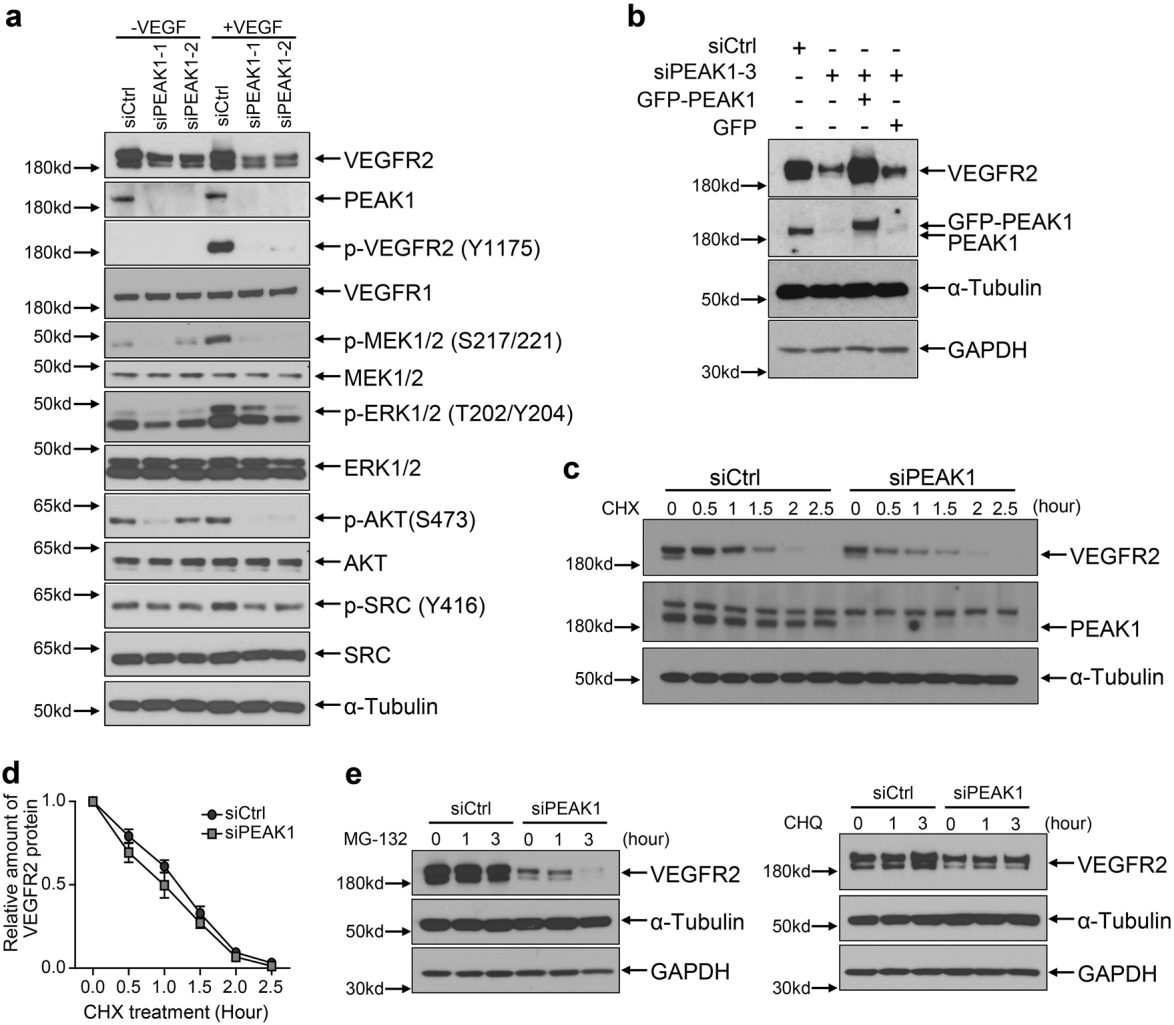
PEAK1 regulates VEGFR2 protein expression and VEGFA-VEGFR2 signaling in human ECs. **a** Western blots (WB) with indicated antibodies of HUVECs treated with siRNAs and stimulated with or without VEGF. In all western blots, α-tubulin and GAPDH served as loading controls. **b** WB of HUVECs treated with siRNAs and transfected with or without non-targeted GFP-PEAK1 or control GFP only. **c** WB of HUVECs treated with siRNAs and with or without protein synthesis inhibitor cycloheximide (CHX) for indicated times. **d** Graph shows relative protein amount of VEGFR2 vs. α-Tubulin of HUVECs treated as in (**c**); Mean ± SEM; *n* = 3. **e** WB of HUVECs treated with siRNAs and with or without proteasome inhibitor MG-132 (left) or lysosome inhibitor chloroquine (CHQ, Right). All data are representative of at least three independent experiments

The VEGFR2 receptor was of major interest because it was ~2.5 fold downregulated in PEAK1-depleted cells (Fig. 4b), and it is the primary receptor tyrosine kinase that mediates VEGFA-induced angiogenesis^2^. Western blotting confirmed the specific downregulation of VEGFR2 protein, but not VEGFR1, in PEAK1-depleted HUVECs (Fig. 5a) and HMVECs (Supplementary Fig. S5). VEGFA ligand activates VEGFR2 and critical downstream signals including MEK/ERK, SRC, and AKT, which can be detected with phosphospecific antibodies that report the activated forms of these proteins^5^. VEGFR2 activation itself and phosphorylation of MEK/ERK, SRC, and AKT were all inhibited in PEAK1-depleted HUVECs compared to control cells upon VEGFA stimulation (Fig. 5a). Similar findings were observed in PEAK1-depleted HMVECs (Supplementary Fig. S5). Importantly, re-expression of GFP-tagged PEAK1 in PEAK1-depleted HUVECs restored VEGFR2 expression (Fig. 5b). Altogether, these findings indicated that VEGFR2 expression and its major downstream signals were severely disrupted in PEAK1-depleted human ECs, which could result in impaired EC proliferation, migration, and survival in response to VEGFA stimuli (Fig. 3).

### PEAK1 regulates *VEGFR2* gene transcription through GATA2

**Fig. 6:**
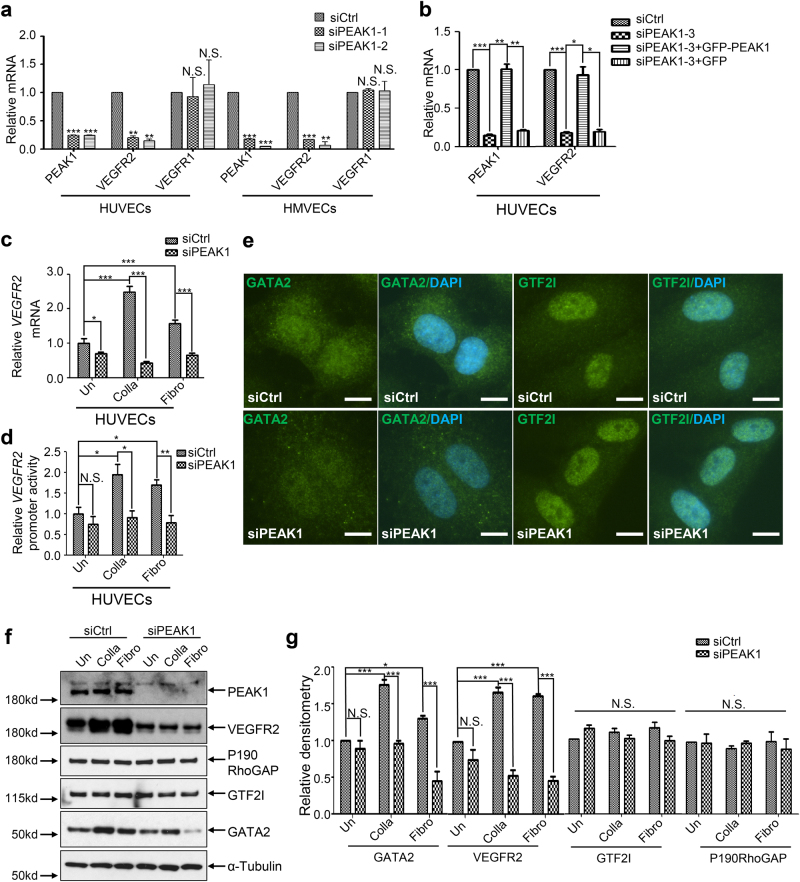
PEAK1 mediates extracellular matrix (ECM)-regulated *VEGFR2* mRNA transcription in human ECs. **a**–**c** Bar graphs represent relative mRNA levels vs. *HPRT1* measured by quantitative RT-PCR (qPCR) and normalized against siCtrl. Mean ± SEM; *n* = 3. **a**, **b** Cells were cultured on collagen I-coated plates. **a** HUVECs or HMVECs were treated with siRNAs. *P* value, vs. siCtrl in each target mRNA. **b** HUVECs were treated with siRNAs and transfected with or without non-targeted GFP-PEAK1 (Rescue) or GFP only; **c** HUVECs were attached to plastic dishes coated with either collagen I (Colla), fibronectin (Fibro), or left uncoated (Un) and treated with different siRNAs. **d** HUVECs treated as in (**c**) were transfected with the plasmid of luciferase driven by VEGFR2 promoter. Bar graph represents relative luciferase activity measured by illuminometer and normalized against siCtrl + Uncoated group. Mean ± SEM; *n* = 4. **e** Confocal images of siCtrl and siPEAK1 treated HUVECs stained with GATA2 or GTF2I antibodies (green) along with DAPI, as a nuclear stain (blue). Cells were cultured on collagen I coated coverglass. Scale bar = 10 μm. **f** WB of HUVECs attached to different coated plates and treated with siRNAs. **g** Bar graph shows relative protein level vs. α-tubulin from HUVECs treated as in (**f**). Relative densitometry from three independent WB were analyzed by Image J. Mean ± SEM; *n* = 3. All data are representative of at least three independent experiments. ***P < 0.001; **P < 0.01; *P < 0.05; N.S., not significant

The expression of VEGFR2 protein in ECs is tightly regulated by proteasome- and/or lysosome-mediated receptor degradation^5^. However, the protein degradation rate of VEGFR2 under the protein synthesis inhibitor Cycloheximide (CHX) treatment was not changed by PEAK1-depletion (Fig. 5c, d). Moreover, neither the proteasome inhibitor MG-132 nor the lysosomal inhibitor Chloroquine (CHQ) rescued the reduced VEGFR2 protein expression caused by PEAK1-depletion, indicating that PEAK1 does not regulate protein degradation of VEGFR2 (Fig. 5e). Since PEAK1-depletion does not alter VEGFR2 protein stability, we investigated whether PEAK1 regulates *VEGFR2* mRNA expression. For these studies, we plated HUVEC cells on collagen I-coated plates since ECM proteins have been reported to upregulate VEGFR2 expression and PEAK1 is a focal adhesion protein that transmits integrin-ECM signals in cancer cells^7, 9, 21^. PEAK1 depletion strongly down-regulated *VEGFR2*, but not *VEGFR1* mRNA levels in both HUVECs and HMVECs (Fig. 6a). Importantly, re-expression of PEAK1 in PEAK1-depleted cells rescued the loss of VEGFR2 mRNA (Fig. 6b) and protein (Fig. 5b). Interestingly, HUVECs adhesion to the ECM protein collagen I or fibronectin dramatically increased *VEGFR2* mRNA expression, and PEAK1-depletion completely abolished this effect (Fig. 6c). Furthermore, the transcriptional activity of the VEGFR2 promoter was also significantly impaired under these conditions (Fig. 6d). These findings indicate PEAK1 is required for ECM-regulated *VEGFR2* gene transcription in ECs.

Mechanical cues induced by EC adhesion to the ECM regulates nuclear localization of GATA2 and GTF2I to regulate VEGFR2 transcription in p190RhoGAP-dependent manner^21^. GATA2 is a transcriptional activator while GTF2I is a transcriptional repressor of *VEGFR2* transcription^21^. Surprisingly, the nuclear-to-cytoplasmic ratios of GATA2 or GTF2I were not altered by PEAK1-depletion in HUVECs attached to a collagen matrix (Fig. 6e and Supplementary Fig. S6a). However, GATA2, but not GTF2I, antibody staining was noticeably reduced in PEAK1-depleted cells compared to control cells, suggesting the GATA2 protein level is specifically down-regulated in these cells. Indeed, GATA2 and VEGFR2, but not GTF2I or p190RhoGAP proteins, were reduced in PEAK1-depleted HUVECs attached to collagen or fibronectin coated plates (Fig. 6f, g). It is also notable that VEGFR2 and GATA2 protein expression were specifically up-regulated in response to HUVEC adhesion to collagen and fibronectin, whereas GTF2I and p190RhoGAP protein expression did not change (Fig. 6f, g). These findings indicate that PEAK1 uniquely regulates VEGFR2 and GATA2 protein expression in response to EC adhesion to the ECM.

**Fig. 7:**
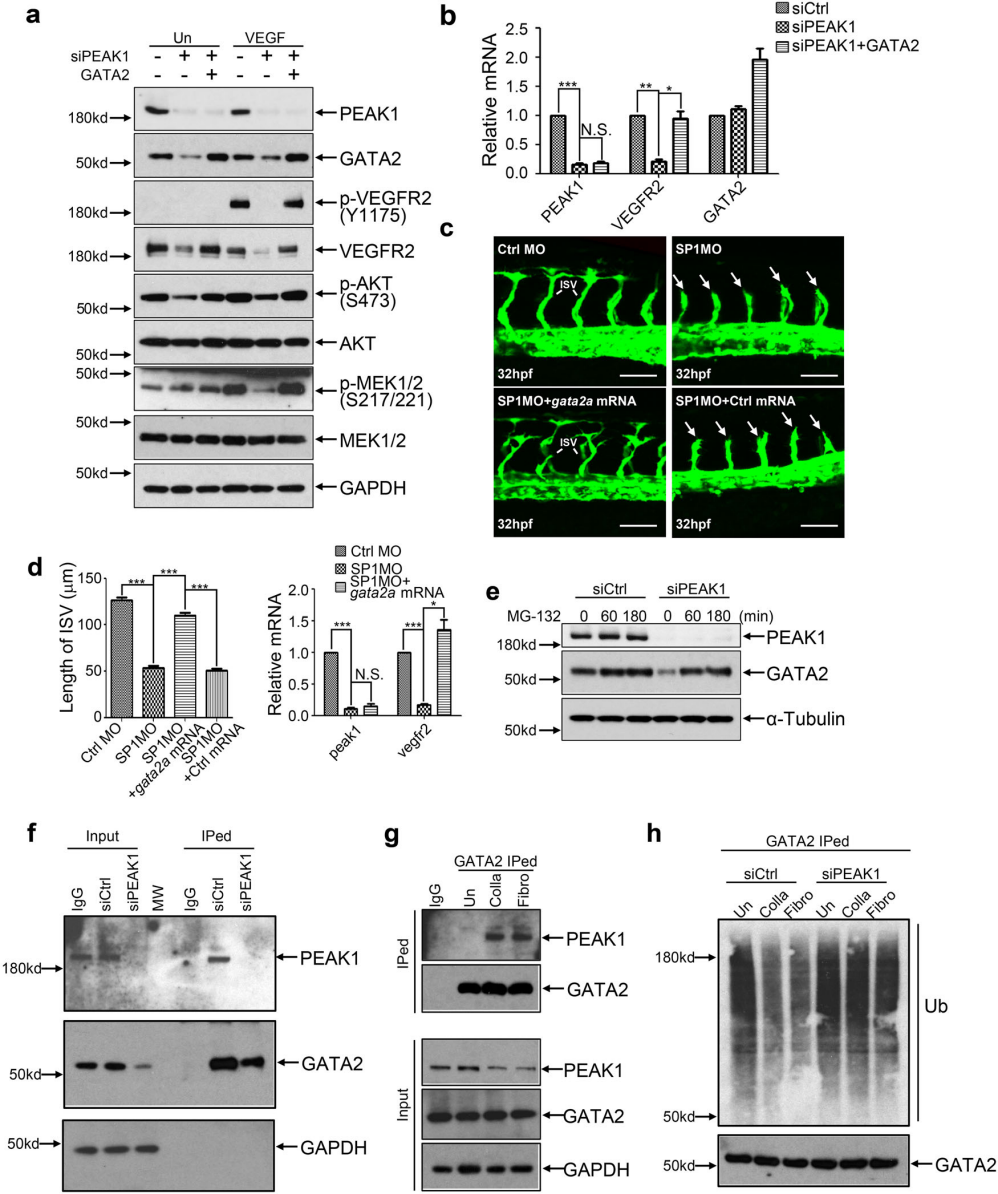
PEAK1 regulates *VEGFR2* transcription through GATA2. **a** WB of HUVECs treated with siRNAs, transfected or not transfected with a plasmid encoding GATA2, and stimulated with or without VEGF for 10 mins. Cells were cultured on collagen I coated plates. **b** Bar graph represents relative mRNA levels vs. *HPRT1* by qPCR analysis of HUVECs treated as in (**a**). **c** Confocal images of ISVs at 32 hpf of *Tg(fli1:egfp)*^*y1*^embryos with co-injection of *gata2a* mRNA or control mRNA and indicated MOs. Arrows point to stunted and disorganized endothelial sprouts. Scale bar = 50 μm. **d** Left bar graph represents the average ISV length of embryos treated as in (**c**). Mean ± SEM; *n* = 50. Right bar graph represents relative mRNA levels vs. zebrafish *actb1* from the embryos treated as in (**c**) by qPCR analysis. Mean ± SEM; *n* = 3. HUVECs were cultured on collagen I-coated plates in (**e**) and (**f**). **e** WB of HUVECs transfected with siRNAs and treated with proteasome inhibitor MG-132. **f**, **g** Co-immunoprecipitated (IP) proteins from HUVECs with anti-GATA2 agarose were analyzed by WB for indicated proteins. In **g**, HUVECs were attached to plastic dishes with indicated coating. To compensate the protein level of GATA2 in Un group, the input was 1.8 folds compared to Fibro and Colla. **h** HUVECs were attached to plastic dishes with indicated coating and treated with different siRNAs. ECs were then treated with MG-132 for 6 h. Ubiquitination of IPed GATA2 protein was analyzed by WB. All data are representative of at least three independent experiments. ***P < 0.001; **P < 0.01; *P < 0.05; N.S. not significant

To determine if PEAK1 regulates *VEGFR2* transcription through GATA2, we re-expressed GATA2 in PEAK1-depleted HUVECs. GATA2 restored *VEGFR2* mRNA and protein expression as well as its downstream signals in human ECs (Fig. 7a, b). Also, because *gata2a* plays a crucial role in zebrafish vascular development by regulating *vegfr2* expression^22, 23^, we tested the ability of gata2a mRNA to rescue the *peak1*-mediated vascular defects in zebrafish. Co-injection of *gata2a* mRNA in *peak1* knockdown zebrafish embryos rescued ISV formation and *vegfr2* mRNA expression (Fig. 7c, d). Moreover, PEAK1 co-immunoprecipitated with GATA2 in an ECM-dependent manner (Fig. 7f, g). Altogether, these findings indicate that PEAK1 associates with GATA2 and that a PEAK1-GATA2 signaling complex works collectively to regulate *VEGFR2* mRNA expression in response to ECM proteins.

Because GATA2 protein, but not mRNA levels, were significantly reduced in PEAK1-depleted ECs (Fig. 6e–g, Supplementary Fig. S6b), and GATA2 protein expression is regulated by ubiquitination^24, 25^, we determined if PEAK1 regulates GATA2 ubiquitination and protein stability. The proteasome inhibitor MG-132 restored the reduced GATA2 protein level in PEAK1-depleted HUVECs (Fig. 7e). Importantly, ECM adhesion inhibited GATA2 ubiquitination and PEAK1 depletion abolished this effect completely (Fig. 7h, Supplementary Fig. S6c). These findings indicate that EC-ECM adhesion promotes the interaction between PEAK1 and GATA2, and regulates GATA2 protein ubiquitination and stability.

### *Peak1* regulates Vegfa-induced angiogenesis in mice

**Fig. 8:**
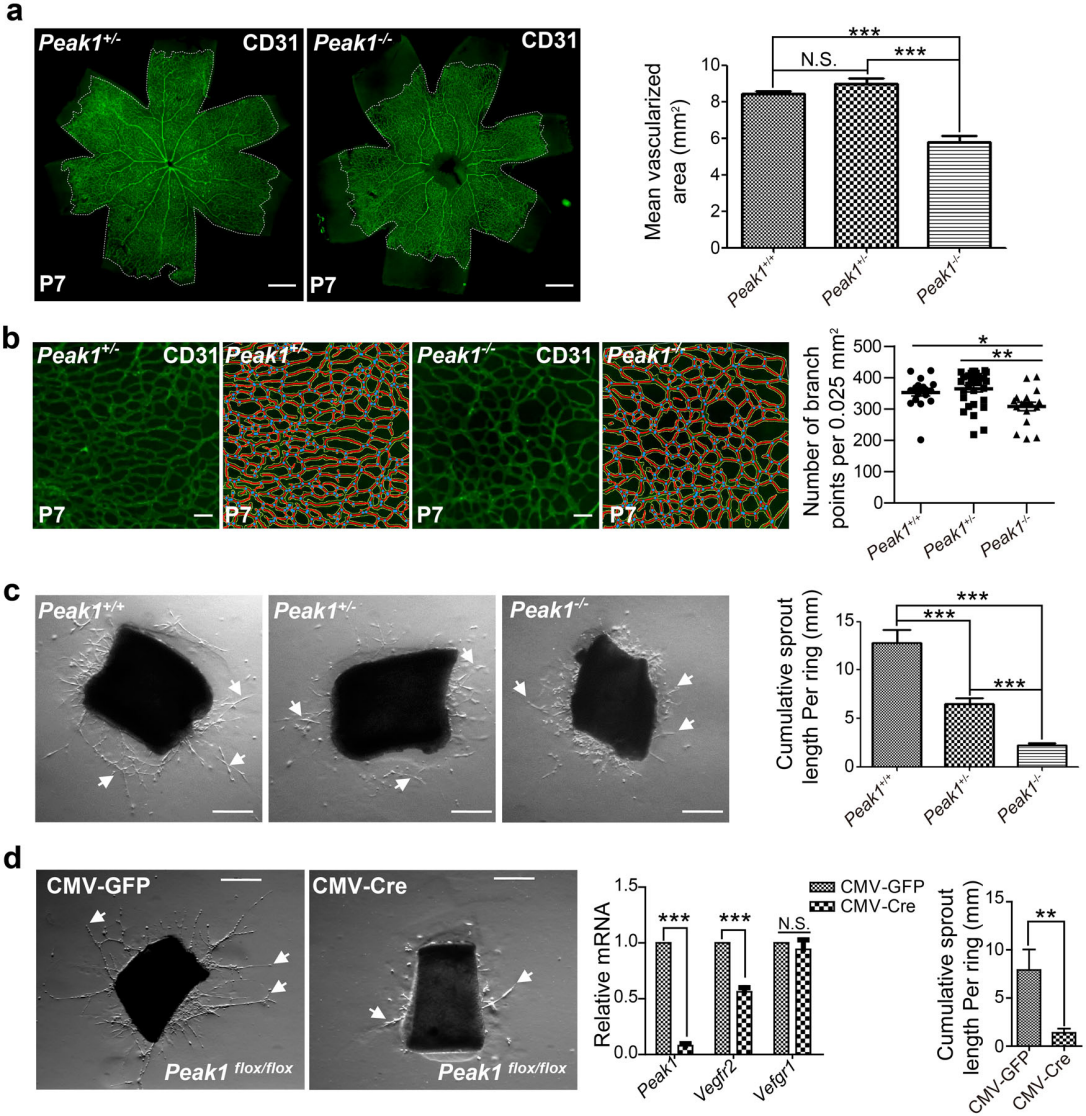
*Peak1* is required for VEGFA-induced angiogenesis in mouse tissues. **a** Anti-CD31 immunofluorescence labeling of the developing vascular plexus of P7 neonatal retinas isolated from heterozygous (*Peak1*^*+/−*^) or homozygous (*Peak1*^*−/*−^) mutant mice. Scale bar = 500 μm. Bar graph represents the mean area of vascularized retinal tissue in *Peak1*^*+/+*^, *Peak1*^*+/*−^, or *Peak1*^*−/*−^ mice; Mean ± SEM; *n* = 6 in *Peak1*^*+/+*^; *n* = 10 in *Peak1*^*+/−*^; *n* = 6 in *Peak1*^*−/*−^. **b** Vasculature branch points of mouse retinas treated as in (**a**) were analyzed by AngioTool. Blue dots indicate branch points. Scale bar = 50 μm. Vertical scatter plot graph represents the average number of branch points per each field. Mean ± SEM; *n* = 18 in *Peak1*^*+/+*^; *n* = 30 in *Peak1*^*+/−*^; *n* = 18 in *Peak1*^*−/*−^. **c** Phase-contrast images of resected aortic tissue embedded in matrigel from *Peak1*^*+/+*^, *Peak1*^*+/−*^ or *Peak1*^*−*/−^ mouse aortas and treated with VEGFA at 37 °C for 6 days. Arrows point to representative vascular sprouts radiating from aortic explants. Scale bar = 300 μm. Bar graph represents the average cumulative length of vessel sprouts per aortic ring; Mean ± SEM; *n* = 10. **d** Phase-contrast images of resected aortic ring tissue embedded in matrigel from *Peak1*^*flox/flox*^ mice and treated ex vivo with adenoviruses encoding either GFP (CMV-GFP) or Cre recombinase (CMV-Cre). The aortic rings were then treated with VEGFA at 37 °C for 6 days. Scale bar = 300 μm. Arrows point to representative sprouts. Left bar graph represents the relative indicated mRNA levels from mouse aortic rings compared to *Hprt1* and normalized with CMV-Cre group. Mean ± SEM; *n* = 3. Right bar graph represents the cumulative vessel length per aortic ring. Mean ± SEM; *n* = 10. ***P < 0.001; **P < 0.01; N.S., not significant

The mouse intraretinal vasculature develops postnatally in a tightly regulated temporal and spatial pattern that can be readily observed by microscopy. In C57BL/6 mice, the superficial vascular plexus forms during the first week after birth by radial outgrowth of vessels from the optic nerve into the periphery, reaching the retinal edges at approximately postnatal day 8 (P8). From P7 onward, the superficial capillaries sprout vertically to form first the deep and then the intermediate vascular plexus. This process is well-known to be ECM, Vegfa/Vegfr2 and Gata2 dependent^21^. Therefore, we wanted to determine the functional relevance of *Peak1* in mouse angiogenesis and retinal plexus formation. To facilitate these studies, we developed *Peak1*^*flox/flox*^ and *Peak1*^*−/−*^ knockout mouse in the C57BL/6 background by targeted deletion of exon 4 of the *Peak1* gene. Genotyping, qPCR, and western blotting of isolated tissues demonstrated that *Peak1*^*−/−*^ is a null mutant (Supplementary Fig. S7a-d and S8b). Interestingly, close examination of developing P7 neonatal retinas from *Peak1*^−/−^ mice revealed significantly reduced outgrowth of the superficial vessel plexus when compared to matched littermates with *Peak1* gene (*Peak1*^*+/−*^, and *Peak1*^*+/+*^) (Fig. 8a). Also, the numbers of branch points of the retinal vasculature in *Peak1* null mutants were significantly reduced (Fig. 8b). These findings indicate that *Peak1* plays an important regulatory role in the angiogenesis of developing mouse retina. These findings are consistent with the findings from human ECs in vitro and developing zebrafish embryos in vivo (Figs. 1 and 5). It should be noted that *Peak1*^*−/−*^ animals bred normally at expected Mendelian ratios, had normal complete blood counts (Supplementary Table S6), and did not show gross developmental or obvious health abnormalities, except for an increased incidence of malocclusions (3.49% in *Peak1*^*−/−*^, *n* = 229 vs. 0.00% in *Peak*^*+/+*^, *n* = 187; while 0.046% in wildtype C57BL/6 mice reported by Jackson Labs), suggesting the phenotype of *Peak1*^*−/−*^ animals are very specific. It is also notable that the vascular plexus in adult retinas from *Peak1*^*−/−*^ and *Peak1*^*+/+*^ mice appeared fully developed without morphological defects (Supplementary Fig. S8a). This suggests that the loss of Peak1 delays retinal vessel formation during early development, but at later stages the animal can compensate for the loss of Peak1 to form a complete and functional retinal plexus.

While it seems *Peak1* is primarily involved in early vascular development of zebrafish and mouse, we investigated whether *Peak1* also plays a role in vascular remodeling processes in adult tissues under certain conditions, such as Vegfa-induced vessel outgrowth in dissected tissues from fully developed adult mice. The ex vivo aortic ring assay is a reliable and reproducible angiogenesis assay to study specific growth factor-induced angiogenesis of mouse tissues in a defined ECM microenvironment^26^. Dissected aortic tissues were cultured in Reduced Growth Factor Basement Membrane Matrix and allowed to spontaneously generate outgrowths of branching microvessels in response to VEGFA treatment, which can be easily monitored and quantified by direct microscopic observation. In this model, aortas from *Peak1*^*−/−*^ and *Peak1*^*+/-*^ mice showed strikingly reduced de novo cumulative vessel formation and reduced number of new vessel sprouts compared to aortas from *Peak1*^*+/+*^ animals (Fig. 8c, Supplementary Fig. S8c). In addition, the average sprout length was dramatically reduced in *Peak1*^*−/−*^ animals compared to *Peak1*^*+/-*^ and *Peak1*^*+/+*^ mice (Supplementary Fig. S8d). We also isolated aortas from *Peak1*^*flox/flox*^ animals and then treated them with an adenovirus either encoding GFP (CMV-GFP) or Cre recombinase (CMV-Cre) to induce de novo *Peak1*-deletion. The CMV-Cre treated aortas showed strongly reduced microvessel formation in response to VEGFA compared to CMV-GFP treated aortas, which was associated with reduced *VEGFR2*, but not *VEGFR1* mRNA expression (Fig. 8d). Altogether, our findings suggest that Peak1 regulates VEGFA-induced angiogenesis by regulating *VEGFR2* mRNA expression in mouse tissues.

### PEAK1 regulates VEGFA-induced angiogenesis in tumor tissues

**Fig. 9:**
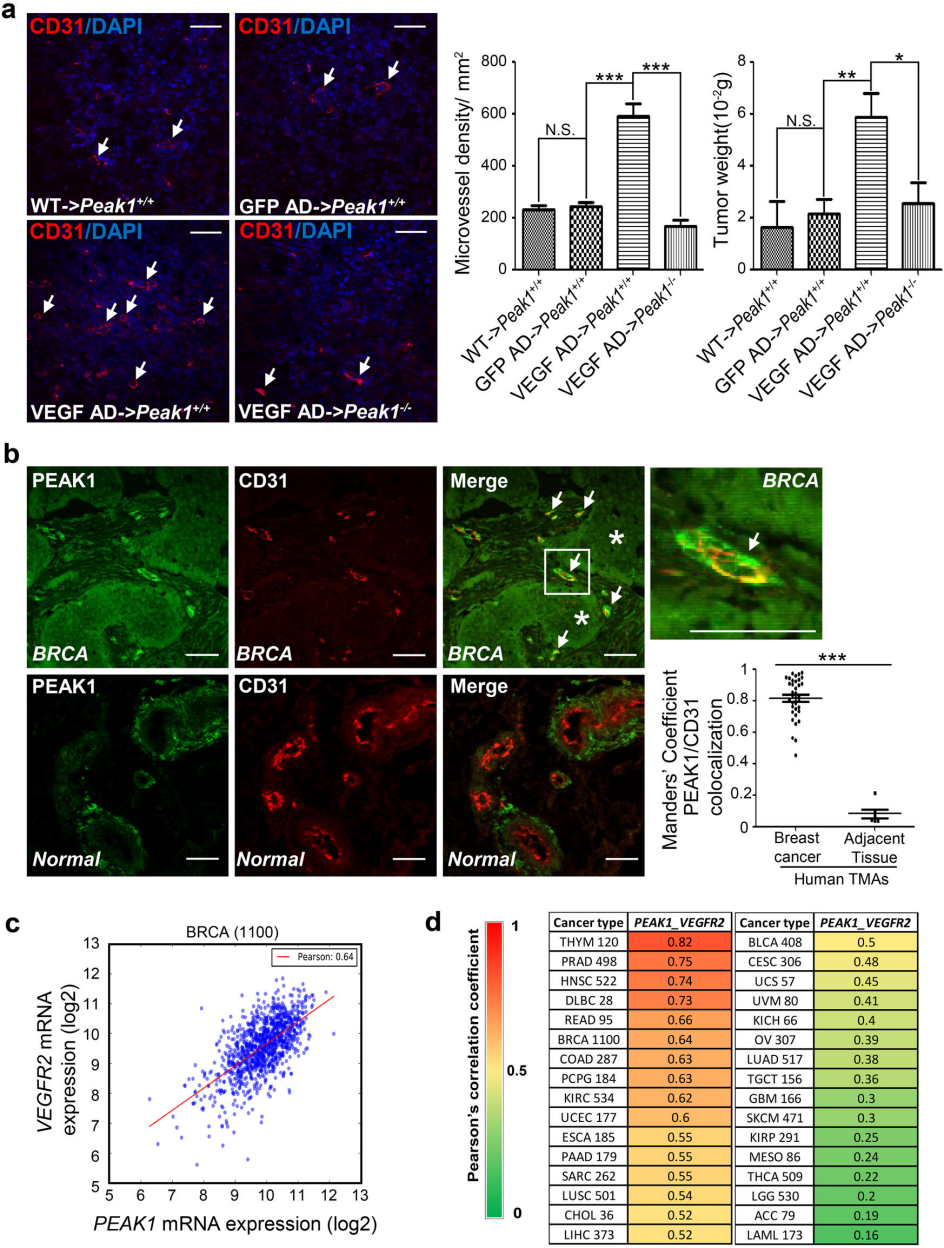
PEAK1 regulates tumor-induced angiogenesis and correlates with VEGFR2 expression in human cancers. **a** Immunofluorescence staining with antibody against CD31 (red) and DAPI (blue) of mouse syngeneic tumor sections at day 10 after injection of tumor cells. WT, original E0771 cells; GFP AD, GFP adenovirus infected E0771 cells; VEGF AD, VEGF adenovirus infected E0771 cells. Arrows point to neo-vessels. Left bar graph represent microvascular density in tumor sections determined by counting the number of CD31-positive vascular structures per square mm. Mean ± SEM; *n* = 18. Right bar graph shows weight of excised tumors. Mean ± SEM; *n* = 6. **b** Confocal images of co-immunofluorescent labeling of CD31 (red) and PEAK1 (green) in breast cancer sections from human tissue microarray. Arrows point to the neo-vessels and stars label the tumor cells. BRCA, breast invasive carcinoma tissue; Normal, adjacent non-cancerous tissue. Vertical scatter plot graph represents Manders’ coefficient analysis of PEAK1 vs. CD31 colocalization. Total of 36 individual cases from tumor tissues and 6 cases from adjacent non-cancerous tissues were analyzed. For each case, three independent fields were imaged by confocal microscope. **c** RNA-seq by Expectation-Maximization (RSEM) normalized RNA-seq data of 1100 breast cancer patients from The Cancer Genome Atlas (TCGA) database were log_2_ transformed and calculated for the Pearson’s correlation coefficient between *PEAK1* and *VEGFR2*. A linear regression fit was also plotted (red line). **d** The heat map shows Pearson’s correlation coefficient between *PEAK1* and *VEGFR2* expression from various human cancer types analyzed as in (**c**). The analyzed case numbers were indicated after the abbreviation of each cancer type. ACC Adrenocortical carcinoma, BLCA Bladder Urothelial Carcinoma, BRCA Breast invasive carcinoma, CESC Cervical squamous cell carcinoma and endocervical adenocarcinoma, CHOL Cholangiocarcinoma, COAD Colon adenocarcinoma, DLBC Lymphoid Neoplasm Diffuse Large B-cell Lymphoma, ESCA Esophageal carcinoma, GBM Glioblastoma multiforme, HNSC Head and Neck squamous cell carcinoma, KICH Kidney Chromophobe, KIRC Kidney renal clear cell carcinoma, KIRP Kidney renal papillary cell carcinoma, LAML Acute Myeloid Leukemia, LGG Brain Lower Grade Glioma, LIHC Liver hepatocellular carcinoma, LUAD Lung adenocarcinoma, LUSC Lung squamous cell carcinoma, MESO Mesothelioma, OV Ovarian serous cystadenocarcinoma, PAAD Pancreatic adenocarcinoma, PCPG Pheochromocytoma and Paraganglioma, PRAD Prostate adenocarcinoma, READ Rectum adenocarcinoma, SARC Sarcoma, SKCM Skin Cutaneous Melanoma, TGCT Testicular Germ Cell Tumors, THCA Thyroid carcinoma, THYM Thymoma, UCEC Uterine Corpus Endometrial Carcinoma, UCS Uterine Carcinosarcoma, UVM Uveal Melanoma. ***P < 0.001; **P < 0.01; *P < 0.05; N.S., not significant. Scale bar = 30 μm

VEGFA/VEGFR2-dependent angiogenesis supports tumor growth in many human cancers^4^. To determine if PEAK1 regulates VEGFA-induced angiogenesis and tumor growth, we developed a syngeneic mouse model of breast cancer using *Peak1*^−*/−*^ null mutant and *Peak1*^*+/+*^ wild type mice and the E0771 breast cancer cell line derived from a spontaneous medullary breast adenocarcinoma in C57BL/6 mice with mutated p53^27^. We also engineered these cells to secrete VEGFA using an adenovirus encoding mouse VEGFA, which drives a strong angiogenic response characterized by increased tumor microvascular density^28^. Indeed, E0771 tumors expressing VEGFA showed significantly increased microvascular density and tumor size in *Peak1*^*+/+*^ animals, compared to tumors derived from control E0771 cells or E0771 cells expressing GFP only. Strikingly, VEGFA-induced tumor growth and microvascular density in *Peak1*^−*/−*^ mice was significantly impaired compared to *Peak1*^*+/+*^ mice (Fig. 9a). These findings indicate that Peak1 expression contributes to VEGFA-driven angiogenesis in syngeneic mouse tumor tissues.

We next determined the vascular expression of PEAK1 in breast cancer patient tissue samples using immunohistochemical staining and a PEAK1-specific antibody^9, 10^. PEAK1 protein was highly expressed and co-localized in CD31-positive vessels in breast cancer tissues compared to adjacent non-cancerous tissues (Fig. 9b). Interestingly, blood vessels in adjacent tissue also showed PEAK1 immunoreactivity, but it was restricted to the abluminal regions of vessels, while in breast cancer samples, it was highly expressed on the luminal side of vessels, where it strongly co-localized with CD31-positive ECs (Fig. 9b). Thus, PEAK1 is highly expressed in ECs of tumor-associated vessels, but not in CD31-positive ECs present in adjacent normal breast tissues.

Using The Cancer Genome Atlas (TCGA) database and bioinformatics, we examined the expression correlations of *PEAK1* mRNA and *VEGFR2* mRNA levels across 32 different human cancers. We found that *PEAK1* and *VEGFR2* mRNA expression are highly correlated in 17 out of 32 cancer types including invasive breast carcinoma (Fig. 9c, d, Supplementary Table S7). Therefore, deregulated expression of the PEAK1-VEGFR2 axis may contribute widely to human cancers. Interestingly, *GATA2* mRNA expression is not significantly correlated with either *PEAK1* or *VEGFR2* mRNA in most cancer types (Supplementary Table S7), which is consistent with our findings that PEAK1 regulates GATA2 protein degradation at the post-translational level (Fig. 7e–h). These findings indicate that Peak1 may play a role in VEGFA/VEGFR2-driven human tumor growth and angiogenesis.

## Discussion

In our study, we investigated the role of PEAK1 in development and angiogenesis using biochemical, proteomic, and in vitro angiogenesis assays combined with established vertebrate animal models of neovascularization. Using these approaches, we provide multiple lines of evidence that a PEAK1-GATA2 transcriptional pathway controls VEGFR2 transcription to drive neovascularization in vertebrates. First, perturbation of *peak1* in zebrafish by MOs or TALENs significantly inhibited angiogenesis and reduced *vegfr2* expression, and most importantly, *vegfr2* expression and vessel development was restored in these animals following co-injection of *gata2a* mRNA (Fig. 7c, d). Second, mechanistic studies in ECs showed that PEAK1 binds to and controls GATA2 protein stability to regulate VEGFR2 transcription and angiogenesis in an ECM-dependent manner. Third, MO-mediated co-depletion of *peak1* and *vegfa* in developing zebrafish synergistically inhibited angiogenesis. Fourth, development of the retinal plexus was significantly delayed in *Peak1*^−/−^ mice, which is largely driven by Vegfa/Vegfr2 signaling^29^, and de novo knockout of *Peak1* in mouse aorta explants robustly inhibited VEGFA-induced new vessel formation, which was associated with reduced VEGFR2 expression. Fifth, mouse breast cancer cells engineered to secrete Vegfa and transplanted into *Peak1*^−/−^ null mice showed significantly impaired in tumor growth and microvascular formation. Finally, interrogation of human cancer databases revealed that PEAK1 and VEGFR2 mRNA expression are highly correlated in many human cancers (17 of 32). Taken together, these findings are consistent with a model in which EC adhesion to the ECM promotes PEAK1 and GATA2 association leading to increased GATA2 protein stability. The elevated GATA2 protein promotes expression of VEGFR2 mRNA and protein, which in turn promotes increased downstream signaling to effectors that drive EC migration, proliferation, survival, and a more proangiogenic phenotype (Supplementary Fig. S9). However, other angiogenic factors also likely contribute to this process. For example, the proangiogenic regulators MMP1, ICAM1, and JAG1 were all significantly downregulated upon PEAK1-depletion in human ECs. On the other hand, PEAK1-depletion in HUVECs did not alter protein expression of other tyrosine kinases including VEGFR1 and EGFR, suggesting PEAK1′s regulation of VEGFR2 is dedicated to this receptor tyrosine kinase. We are currently investigating how PEAK1 coordinates the regulation of these angiogenic factors and the role that the ECM plays in this process.

While our findings indicate that PEAK1-GATA2 signaling regulates VEGFR2 transcription in response to ECM signals, it is likely that VEGFR2 transcription is tightly regulated by multiple signaling networks that impinge on nuclear transcription factors such as GATA2^30^, GTF2I^31^, NF-κB^32^, Sp1^33^, HIF-1α^34^, HESR-1^35^, and Stat3^36^. For example, changes in ECM elasticity control nuclear localization of GATA2 to mediate VEGFR2 transcription in ECs^21^. Nuclear GATA2 levels were higher in cells attached to stiffer gels and VEGFR2 mRNA and protein were increased under these conditions^21^. This response was dependent on p190RhoGAP signaling to mediate VEGFR2 transcriptional upregulation. Our findings reveal another distinct level of regulation of VEGFR2 transcription induced by the ECM. We found that GATA2 protein levels are modulated by ubiquitination and proteasome destruction in response to EC adhesion to the ECM. PEAK1 and GATA2 were observed to co-precipitate in ECs in response to ECM adhesion, which suggests that PEAK1 may shield GATA2 from the ubiquitination machinery leading to increased protein stability. This in turn could increase the effective concentration of GATA2 available to activate VEGFR2 transcription. GATA2 is known to be ubiquitinated by E3 ubiquitin ligase SCF^FBW7^ (a complex of SKP1, cullin-1, and FBW7) in HEK293 and Hela cells^24^, and FBW7 is required for proper angiogenesis in mice^37^. Interestingly, it appears that PEAK1-GATA2-mediated VEGFR2 expression operates independent of GTF2I and p190RhoGAP as we did not observe changes in their protein expression nor did we observe changes in nuclear localization of GTF2I in response to PEAK1 signaling. It should be noted that our studies were performed using ECM-coated dishes which are noncompliant rigid matrices, whereas the study by Mammoto and colleagues were performed using compliant elastic gels. These findings support the emerging idea that VEGFR2 expression is tightly regulated by multiple pathways driven by differences in ECM elasticity and mechanosensing cues present in the extracellular environment.

While it has been shown that PEAK1 has weak tyrosine kinase activity in vitro^7^, its putative endogenous substrate has yet to be identified. Recent evidence indicates that the putative ATP binding site of PEAK1 is occluded in its crystal structure^38^, suggesting it may act as a pseudo-kinase in physiological conditions. Moreover, previous studies indicates that tyrosine phosphorylation of PEAK1 depends on SRC family kinases activity^7, 9, 10, 39^, while we found that SRC activity is dispensable for PEAK1′s regulation of VEGFR2 transcription in ECs (data not shown). We have also analyzed phosphorylation sites of human GATA2 with NetPhos 3.1 server and identified 11 putative tyrosine phosphorylation sites; however, none of these sites have been related to the transcriptional regulation of VEGFR2. Taken together, our results support the hypothesis that PEAK1 acts as a scaffold protein rather than a kinase in ECs where it interacts with GATA2, and this interaction inhibits GATA2 ubiquitination and increased its protein stability, which in turn increases VEGFR2 mRNA expression.

Besides the delayed development of the retinal vasculature and malocclusions in mice, we did not observe gross defects in overall body and organ development in *peak1*-null zebrafish or in *Peak1*^*−/−*^ mice. Homozygous *Peak1*^*−/−*^ mice were born at expected Mendelian ratios, appeared normal into adulthood, were fertile, and showed only a slightly increased incidence of malocclusions. Also, *peak1* null zebrafish embryos (homozygous *peak1*^*∆2/ ∆2*)^ did not display vascular defects and survive to become fertile adults (data not shown). These findings indicate that Peak1 could be largely dispensable for normal embryonic development. However, genetic compensation due to deleterious gene mutations is likely at play in these animals. Indeed, treatment of *peak1*^*∆2/ ∆2*^ null zebrafish embryos with peak1 specific MOs failed to induce defects in embryogenesis and vascular formation indicating that these animals had activated functional compensatory mechanisms^16^. Genetic compensation induced by deleterious gene mutations, but not gene knockdown, commonly lead to different phenotypes as recently described for egfl7 mutants and MO-induced morphant zebrafish^16^. While the mechanisms underlying the compensation in Peak1 mutants are likely complex, initial findings in our laboratory indicate that Pragmin (SGK223) is an unlikely candidate even though it is PEAK1′s closest relative and the only other member of the NKF3 family of tyrosine kinases^6^. In fact, Pragmin protein expression in *Peak1*^*−/−*^ MEFs and *Pragmin* mRNA levels in *Peak1*^−/−^ mouse tissues are not altered (data not shown). Also, PEAK1 knockdown in HUVECs did not alter Pragmin expression. However, to fully address this issue it will be necessary to knockout both PEAK1 and Pragmin in zebrafish and mice.

Although Peak1 compensatory mechanisms may operate during animal development, our findings clearly demonstrative that Peak1 plays a critical role in mediating angiogenesis in adult organisms. Angiogenesis was strongly impaired in aortas isolated from *Peak1*^−/−^ adult mice and tumor-induced angiogenesis was impaired in *Peak1*^−/−^ mice. Also, angiogenesis was strongly inhibited in aortas isolated from *Peak1*^*flox/flox*^ mice and treated with adeno-Cre to delete the *Peak1* gene. These findings indicate that PEAK1 plays a more prominent role in adult animals in response to stress conditions such as tissue injury and cancer. In fact, in normal non-cancerous vessels, PEAK1 expression was specifically restricted to the outer abluminal surface of mature vessels, but not the endothelium. These findings suggest that PEAK1 may be up-regulated in newly forming vessels and down-regulated during vessel maturation. It should also be noted that PEAK1 expression is elevated in many human cancers including breast and pancreatic^9–11, 39, 40^. More interestingly, recent study suggested the treatment of an anti-VEGFA monoclonal antibody in pancreatic ductal adenocarcinoma lead to SRC kinase-induced phosphorylation of PEAK1 in a collagen-dependent manner^41^. These findings suggest a possible role of PEAK1 in resistance to anti-VEGFA therapies, which are the main therapies used to target tumor-induced angiogenesis in clinic^42^. The fact that PEAK1 drives both tumor progression and mediates tumor-induced angiogenesis makes it a promising therapeutic target for cancer and other angiogenesis-dependent disorders.

## Materials and methods

### Animals

All animals were treated according to the University of California San Diego animal welfare guidelines as described and approved by the UCSD Institutional Animal Care and Use Committee (S12005 and S06008). Wildtype AB, transgenic *Tg(fli1:egfp)*^*y1*^ and *Tg(fli1:nls-egfp)*^*y7*^ zebrafish were kindly provided by Dr. David Traver (UCSD). Zebrafish were maintained as previously described^43^. C57BL/6 J mice were purchased from Jackson Laboratory. All mice were maintained in a specific pathogen-free vivarium.

### Generation of *Peak1*^*flox/flox*^ mice and *Peak1*^*−/−*^ mice

*Peak1*^*flox/flox*^ mice were generated by Ingenious Targeting Laboratory. Briefly, targeted iTL IC1 (C57BL/6) embryonic stem cells were microinjected into Balb/c blastocysts, from which chimeras with a high percentage of black coat color were crossed with C57BL/6 FLP mice to remove the Neo cassette. Resulting heterozygous *Peak1*^*flox/+*^ mice were crossed with *Tg(EIIa-Cre)* C57Bl/6 mice (kindly provided by Dr. Jeffrey D. Esko, UCSD) to generate heterozygous *Peak1*^*+/−*^ mice, which were then in-crossed to produce the homozygous *Peak1*^*−/−*^ straight knockout mice. Mice were genotyped using the schematic strategy (Supplementary Fig. S7a) with primers listed in Supplementary Table S1.

### Generation of heritable TALEN-mediated mutations in zebrafish *peak1* gene

TALEN plasmids targeting zebrafish *peak1* gene were constructed from the stock cassette as previously published^14^ and the targeting site was illustrated in Supplementary Fig S2b. TALEN mRNAs were synthesized with Sp6 transcription kit (Ambion) as described in online methods. TALEN mRNAs of the left arm (TALEN L) and the right arm (TALEN R) were mixed at a 1:1 ratio to a final concentration of 60 ng/μl each. A total of 1 nl of TALEN mRNAs was injected into the one-cell stage embryos. Genomic DNA was extracted from individual embryo or from the tail fin of adult zebrafish and was genotyped by PCR and MslI enzyme digestion, and confirmed by sequencing as previously described.^14^

### Cell culture

Human Umbilical Vein Endothelial Cells (HUVEC, C2517A) and Human Cardiac Microvascular Endothelial Cells (HMVEC-C, CC7030) were purchased from Lonza and cultured in EGM-2 medium (Lonza) on collagen I (5 µg/cm^2^, Gibco, A10483) or fibronectin (1 µg/cm^2^, Sigma, F1141) pre-coated plates unless otherwise indicated. Cells obtained from Lonza were considered as passage 1, and only cells from passage 3 to 6 were used for the experiments. Cells were harvested for analysis at sub-confluent stage. E0771 murine breast cancer cells were purchased from CH3 Biosystems and cultured in RPMI-1640 medium (Gibco) with 10% fetal bovine serum (FBS, Gibco). Primary endothelial cells and E0771 cells were authenticated by the company. All cell stocks were tested for mycoplasma contamination by PCR and were negative.

### Morpholino and microinjection

Morpholino antisense oligonucleotides (MOs) were purchased from Gene Tools (Philomath, OR). SP1MO 5′-GACTGAGAAAGGTAGCTTACTTCAT-3′ and SP2MO 5′- AGATCTGAGA TCAGACAAAACGGGA- 3′ were used to block zebrafish *peak1* gene, VEGFMO 5′-GTATCAAATAAACAACCAAGTTCAT-3′ was used to block zebrafish *vegfa* gene^18^, CtrlMO 5′-CCTCTTACCTCAGTTACAATTTATA-3′ was used as a control morpholino. Unless otherwise indicated, 3.2 ng MOs were injected with 0.1% phenol red into zebrafish embryos at the 1 cell stage.

### In vitro transcription, mRNA injection and rescue experiments

The coding region of zebrafish *peak1* gene was cloned into pCS2^+^ vector for zebrafish *peak1* mRNA synthesis, while pCSDest-gata2a-2A-tdTomato plasmid was kindly provided by Dr. David Traver for zebrafish *gata2a* mRNA synthesis. The mMESSAGE mMACHINE® SP6 Transcription Kit (Thermo Fisher) was used to synthesize the mRNA by in vitro transcription. After precipitation with lithium chloride (in the kit) and washing with 70% pre-chilled ethanol for at least 3 times, the mRNA was dried and resuspended in RNase free water and the concentration was determined by Nanodrop (Thermo Fisher). Rescue experiments were conducted by co-injection of 100 pg *peak1* mRNA, *gata2a* mRNA or 100 pg control RFP mRNA with 3.2 ng indicated MO into zebrafish embryos at the 1 cell stage.

### Zebrafish cell transplantation assay

Cell transplantation was performed, as described previously^44^. Briefly, *Tg(fli1:EGFP)*^*y1*^ embryos were used as the donors and wildtype AB embryos were used as recipients. At the one-cell stage, either the donor embryos or the recipient embryos were injected with the indicated morpholino (Supplementary Fig. S3). And phenol red was injected into donor embryos as an indicator. At the sphere stage, embryos were manually dechorionated. Approximately 40–60 cells from the marginal zone of a donor embryo were transferred to the counterpart region of a recipient embryo. The recipient embryos were then grown at 28 °C and imaged at 32hpf. Endothelial cells (ECs) in chimeric embryos originating from donor *Tg(fli1:EGFP)*^*y1*^ embryos were visualized by their green fluorescence under the Olympus FV1000 confocal microscope. The length of each fluorescent intersegmental vessels (ISV) was measured using the Nikon Elements software.

### SiRNAs and transfection

Predesigned siRNAs were purchased from Sigma or Qiagen. The catalog numbers of all the siRNAs were listed in the Supplementary Table S3. siPEAK1 is a 1:1 mixture of siPEAK1-1 and siPEAK1-2. HUVECs, HMVECs, U87 or U87 EGFRVIII cells were transfected with indicated siRNA using lipofectamine RNAiMAX (Invitrogen) according to the manufacturer’s manual. The knockdown efficiency of these siRNAs were confirmed by qPCR and western blot. All the primers used in this study were listed in Supplementary Table S1.

### Plasmids and transfection

The plasmid pcDNA-GATA2 (#1287) and pGL3-VEGFR2-780 (#21307) were purchased from Addgene and amplified and purified with endotoxin free mini plasmid prep kit (MO BIO Laboratories). The purified plasmids were transfected into HUVECs using Lipofectamine 3000 (ThermoFisher Sci) according to manufacturer’s protocol.

### Fibrin gel angiogenic sprouting assay

Angiogenic sprouting assay was performed, as previously described^45^. Briefly, Cytodex 3 beads (GE healthcare) were coated with HUVECs at a concentration of 400 cells per bead at 37 °C for 4 h and then allowed for adhesion overnight in EGM-2 medium. HUVEC-coated beads were resuspended in 2 mg/mL fibrinogen solution (Sigma) at a concentration of 500 beads/ml. In total, 500 μl (0.625 Units/mL) of thrombin was added to each well of a 24-well plate, then 500 μl of fibrinogen/bead suspension was added to each well. After gels clotted, fibroblasts were seeded on top of the gel at a concentration of 20,000 cells per well. Beads were photographed at Day 5 with bright-field microscopy and analyzed in the Nikon Elements software.

### Matrigel based tube formation assay

Tube formation assay was performed as described^46^. Briefly, HUVECs were seeded in matrigel (growth factor reduced basement membrane matrix, Trevigen) and incubated with EGM-2 medium or EBM-2 medium (Lonza) with 2% FBS and 100ng/ml VEGF (Peprotech). Cells were incubated at 37 °C for 6 h, fixed in 4% PFA and imaged with bright-field microscopy. Five randomized fields were taken per each condition in each experiment. The cumulative length of the tubes were measured using the Nikon Elements software.

### Real time cell analysis: cell migration and proliferation

Experiments were carried out using the xCELLigence RTCA DP instrument (ACEA Biosciences) in a humidified incubator at 37 °C and 5% CO2 according to manufacturer’s protocols. Cell proliferation experiments were performed on collagen-coated 16-well E plates (ACEA Biosciences). SiRNA transfected ECs suspended in EBM-2 + 0.5% FBS were plated at 1500 cells/well after overnight starvation. The impedance of each well surface, which indicates the cell spreading area, was recorded at 15-min intervals as arbitrary units (Cell Index), and plotted against time to generate cell proliferation curves for a 72 h period. The proliferation slopes (Cell Index/hour) of the curves from 3 h to 72 h after plating were calculated using the xCELLigence software. Cell migration experiments were performed on 16-well CIM plates (ACEA Biosciences) with both sides of the upper chamber coated with Collagen. SiRNA transfected ECs suspended in EBM-2 + 0.2% FBS were plated at 30,000 cells/well after overnight starvation. The bottom chamber was loaded with EBM-2 + 2%FBS with or without 30ng/ml VEGF. The impedance of the bottom surface of the upper chamber, which indicates the presence of the migrated cells, was recorded at 15-min intervals as arbitrary units (Cell Index), and plotted against time to generate cell migration curves for a 16 h period. The migration slopes (Cell Index/hour) of the curves from 2 to 12 h were calculated by the xCELLigence software. All experiments were done in quadruplicates.

### Annexin V/7-AAD assay for apoptotic analysis of HUVECs

Briefly, HUVECs were transfected with siRNAs and cultured in EBM-2 + 0.5% FBS medium with or without 50ng/ml VEGF for 24 h. After trypsinization, apoptotic rate of HUVECs was measured by staining cells with FITC Annexin V Apoptosis Detection kit with 7-AAD (Biolegend) followed by fluorescence-activated cell sorting (FACS) analysis using FACSCanto (BD). Cells that were Annexin positive and 7-AAD negative (early) or positive (late) were designated as apoptotic. Pan Caspase inhibitor Z-VAD-FMK (R&D system) was applied at a concentration of 100μM to block caspase-dependent apoptosis.

### Quantitative proteomic analysis of HUVECs with TMT labeling and mass spectrum

Cells were lysed in in 50 mM HEPES (Sigma), pH 8.5, containing 75 mM NaCl (Sigma), 3% sodium dodecyl sulfate (Fisher), 1 mM sodium fluoride (Sigma), 1 mM beta-glycerophosphate (Sigma), 1 mM sodium orthovanadate (Sigma), 10 mM sodium pyrophosphate (Sigma), 1 mM phenylmethylsulfonyl fluoride (Sigma) and 1 × Complete mini EDTA free protease inhibitors (Roche)^47^. Proteins were digested in a two-step process with LysC (Wako) and Trypsin, and then desalted with C18 Sep-Paks (Waters) as previously described^48^. Samples were labeled with 10-plex TMT reagents (Thermo Scientific) as previously described^49^. Fractionation was carried out by basic pH reverse-phase liquid chromatography with fraction combining, as previously described^48^. LC-MS2/MS3 experiments were conducted on an Orbitrap Fusion (Thermo Fisher) with an in-line Easy-nLC 1000 (Thermo Fisher). The Orbitrap Fusion was run in data-dependent mode, where a survey scan was collected over 500–1200 m/z at a resolution of 120,000 in the Orbitrap. For MS2/MS3 analysis, the decision tree option was used, with charge state and m/z range as qualifiers. MS3 analysis was conducted using the synchronous precursor selection (SPS) option to maximize TMT quantitation sensitivity^50^. Centroided data were collected for all MS3 scans. Resultant data files were processed using Proteome Discoverer 2.1 (Thermo Fisher). MS2 data were queried against the Uniprot Human database using the Sequest algorithm^51^. Reporter ion intensities from TMT reagents were extracted from MS3 spectra for quantitative analysis, and signal to noise values were used for quantitation. Protein level quantitation values were calculated by summing signal to noise values for all peptides per protein meeting the specified filters. Data were filtered to a 1% peptide and protein level false discovery rate using the target-decoy strategy^52^. Data were normalized in a two-step process, whereby they were first normalized to the mean for each protein. To account for variation in the amount of protein labeled, values were then normalized to the median of the entire dataset. Final values are reported as normalized summed signal to noise per protein per sample. Gene ontology analysis was conducted on proteins that were significantly differentially regulated (*p* < 0.05, student’s t-test, fold-change ≥ 2) between samples using the DAVID server^53, 54^. All identified proteins were specified as the background. Filters were set as previously described^55^. Significant associations (Benjamini-Hochberg *p* < 0.05) with a false discovery rate of less than 5% are reported.

### Quantitative RT-PCR

Total RNA was extracted from cultured cells or zebrafish embryos with Trizol (Invitrogen) and purified with the RNeasy RNA purification kit (Qiagen). cDNA was synthesized with iScript cDNA synthesis kit (Bio-Rad), and quantitative RT-PCR was performed on StepOnePlus real time PCR system (Applied Biosystems) with SYBR Green probes (Applied Biosystems). Human or mouse hypoxanthine phosphoribosyltransferase 1 (HPRT1) gene or zebrafish beta-actin1 (*actb1*) gene was used as an internal reference respectively. Ratios of the expression level of each gene to the reference gene were then calculated with Microsoft Excel. Primers used in this study were listed in Supplementary Table S1.

### Immunoblots and co-immunoprecipitation

ECs were cultured and treated with siRNAs or plasmids as described above. Cells were harvested at sub-confluent stage. For VEGF stimulation, cells were transfected with indicated siRNA for 48 h, then starved for 12 h in EBM-2 medium with 0.5% serum, and stimulated with 50ng/ml VEGF for 10 min. For MG-132 and Chloroquine treatment, 10 μM MG-132 (Sigma) and 100 μM Chloroquine (Sigma) were used respectively to treat the HUVECs for indicated times. Cell lysates were generated using standard methods with lysis buffer (50 mM HEPES, pH 7.4, 150 mM NaCl, 1% Triton X-100, 1% sodium deoxycholate, 0.1% SDS, 10% glycerol, cocktail protease inhibitors and phosphatase inhibitors). For co-immunoprecipitation, cell lysates from 15 cm plates were 1:1 diluted with HNTG buffer (50 mM HEPES, pH 7.4, 150 mM NaCl, cocktail protease inhibitors and phosphatase inhibitors). A 30 μl agarose slurry of normal mouse IgG-AC (Santa Cruz) or mouse anti-GATA2 AC (Santa Cruz) was added to the cell lysates respectively. For analyzing the poly-ubiquitination of GATA2, HUVECs were cultured and treated with siRNAs as described above and then treated with 10μM MG-132 for 6 h. Cells were then lysed with Lysis buffer II (50 mM HEPES, pH 7.4, 150 mM NaCl, 1% Triton X-100, 1% sodium deoxycholate, 0.5% SDS, 10% glycerol, cocktail protease inhibitors and phosphatase inhibitors) for 30 min. A 30 μl agarose slurry of mouse anti-GATA2 AC (Santa Cruz) was then added to the cell lysate and incubated for 3 h. Immunoprecipitated GATA2 were then washed with Lysis buffer II for three times and boiled with sample buffer for western blot. For immunoblots analysis, proteins were boiled in LDS sample buffer and resolved on Nupage 4–12% gel (Invitrogen). All the antibodies used in this study were listed in Supplementary Table S2.

### Immunofluorescence labeling of cells

HUVECs were cultured on collagen-coated coverglass as indicated above. The cells were then transfected with siRNAs as described above. After 48 h of transfection, the cells were fixed with fresh 4% paraformaldehyde and permeabilized with 0.2% triton in PBS. The cells were then stained with indicated antibodies listed in Supplementary Table S2 and DAPI, as previously described^56^. Fluorescent images of 10 independent fields of each sample were taken by Nikon Eclipse Ti epi-fluorescence microscope and analyzed using the Image J 1.50b software with JACOP plugin.

### Luciferase reporter assay

Luciferase reporter assay was performed, as previously described^21^. Briefly, plasmid pGL3-VEGFR2-780 (Addgene) was transfected into HUVECs with lipofectamine 3000 (Thermo Fisher). After 12 h, siRNAs were transfected as described above. The expression of luciferase was assayed using Luciferase Assay System kit (Promega), and the luciferase activity was measured in duplicates in SpectraMAX Gemini EM (Molecular Devices). Four biological replicates were done for each sample and non-transfected cells were used as blank control.

### Overexpression of GFP-PEAK1 in HUVECs by lentivirus infection

Briefly, high titer lentiviruses (10E8 plaque-forming unit (PFU)/ml) encoding GFP-PEAK1 gene and control GFP gene were purchased from Sirion Biotech (Germany). Transduction was performed in cell pellet with 25 μl of indicated virus after washing with PBS and SureEntry reagent (1:1250, Sabiosciences). 48 h after transduction, cells were selected with Puromycin (1 μg/mL) for 7 days. Surviving cells were used for further experiments.

### Isolation of mouse embryonic fibroblasts

Mouse fibroblasts were isolated from skin punch of E18 week *Peak1*^*+/+*^ and *Peak1*^*−/*−^ mouse embryos. Briefly, pregnant mice were euthanized with CO_2_ and cleaned with ethanol before embryos were extracted. Embryo tissue explants were washed with sterile PBS, containing 1% penicillin/streptomycin, transferred to the plastic plate and covered with medium. Fibroblasts that started to appear on the border of the tissue 5 to 7 days later were expanded and used in experiments.

### Whole mount immunofluorescent staining of the neonatal mouse retina

Whole mount immunofluorescent staining of mouse retina was performed as previously described^57^. Briefly, retinas harvested from postnatal P7 pups and littermates were fixed in 4% paraformaldehyde (PFA) for 10 min and then dissected in PBS. The retinas were then permeabilized in pre-chilled methanol for 10 min and blocked in PBS with 20% FBS/20% normal goat serum (NGS) + 0.3% Triton X-100 for 2 h. The retinas were incubated with primary antibodies in (10% FBS/10% NGS) + 0.3% Triton X-100 in PBS) overnight (O/N) at 4 °C. After extensive washing, the retinas were incubated with secondary antibodies for another 2 h at room temperature (RT). In Fig. 8b, c, two groups of littermates were used. All the antibodies were listed in Supplementary Table S2. Fluorescent image of the whole retina was taken by Keyence BZX-700 Fluorescent Microscope and analyzed using the Nikon Elements software. The branch points of the vasculature from 3 independent fields from each retina were analyzed by the AngioTool^58^ downloaded from National Cancer Institute website.

### Mouse aortic ring assay

The aortic ring assay was performed, as described previously^26^. Thoracic aortae were isolated from 8 weeks old *Peak1*^*−/*−^ mice or the *Peak1*^*+/+*^ and *Peak1*^*+/*−^ littermates. Thoracic aortae were then cleaned and sliced into 0.5 mm long rings. Or thoracic aortae were isolated from 8 weeks old *Peak1*^*flox/flox*^ mice and were infected with adenoviruses (CMV) encoding either GFP or Cre recombinase (Viral Vector Core Facility, University of Iowa Carver College of Medicine). After overnight starvation in the Opti-MEM medium (Invitrogen), aortic rings were placed in wells of a 48-well plate containing 65μl solidified Cultrex® Reduced Growth Factor Basement Membrane Matrix (Trevigen) and then covered with additional 65μl Matrigel. After incubation at 37 °C for 30 min, each well was filled with Opti-MEM medium supplemented with 2.5% FBS and 30ng/ml VEGFA. Rings were incubated at 37 °C for 6 days, and medium was changed every 2 days. The rings were photographed with a Leica M165 FC stereomicroscope and images were analyzed using the Nikon Elements software.

### Mouse syngeneic tumor model

E0771 cells were infected with mouse VEGFA adenovirus (Cell Biolabs) or GFP control adenovirus at multiplicity of 100, as previously described^28^. Unselected population of virus-infected or non-infected cells (1E6 cells/100 μl) were subcutaneously injected into the bilateral flanks of immunocompatible 8-week old *Peak1*^*+/+*^ or *Peak1*^*−/−*^ female mice. The mice were euthanized after 10 days, and the tumors were isolated, weighed and fixed in 4% PFA overnight. The fixed tumors were then sent to the histology core of the Moores Cancer Center (UCSD) for routine paraffin embedding and sectioning.

### Immunofluorescence labeling of human tumor tissue microarrays and mouse tumor sections

Immunofluorescence labeling of human tumor tissue was performed, as previously described^59, 60^. Antigen retrieval was done with 0.5 M Tris–HCl buffer (pH 10) for 10 min in a pre-heated pressure cooker (Nordic Ware). Antibodies were diluted in TBS buffer with 5% NGS and 1% BSA. All the antibodies were listed in Supplementary Table S2. Sections were incubated overnight at 4 °C with primary antibodies. After extensive washing, sections were then incubated for 2 h at RT with fluorescently labeled secondary antibodies. Negative controls were conducted with the particular isotype controls. Human breast cancer tissue microarrays were purchased from US Biomax (BR8014 and BR243). Stained sections were imaged in Olympus FV1000 confocal microscope and analyzed using the ImageJ 1.50b software with JACOP plugin.

### Pearson’s correlation coefficient analysis of mRNA expression in cancer patients

RSEM^61^ normalized RNA-seq data of 9283 TCGA patients spanning 32 different cancer types were downloaded from Firehose (https://confluence.broadinstitute.org/display/GDAC/Home) on Aug 21, 2015. We then log_2_ transformed the RNA-seq data and calculated the Pearson’s correlation coefficient between the mRNA expression of *PEAK1* and *VEGFR2* in each cancer type. A linear regression fit was plotted and overlaid with scatter plot for visualization.

### Data availability

All data are available within the article and its Supplementary Information files or from the corresponding author upon reasonable request.

### Statistical analysis

Sample size of mouse syngeneic model was estimated by using statistical power (biomath http://www.biomath.info/power/ttest.htm). Briefly, with a sample size of 6 tumors, significant differences of 30% can be detected with 80% power at a 0.05 significance level, assuming a standard deviation within groups of 0.15. GraphPad Prism5 were used to perform the statistical analysis (GraphPad software). The data distribution was assumed to be normal, although we did not formally test it. Results are presented as Mean ± SEM. Statistical significance was determined by two-tailed Student’s *t* test, One-way or Two-way ANOVA with Bonferroni post-test respectively. ***P < 0.001; **P < 0.01; *P < 0.05; (N.S.), not significant.

## Electronic supplementary material

Supplementary Figures(PDF 1571 kb)

Supplementary Tables(DOCX 263 kb)

Supplementary Table S4(XLSX 995 kb)

Supplementary Table S5(DOCX 23 kb)

Supplementary Movie S1

Supplementary Movie S2
