# Supplementary material for: Pseudopodium-enriched atypical kinase 1 mediates angiogenesis by modulating GATA2-dependent VEGFR2 transcription
Source: Cell Discov. 2018 May 29;4:26. doi: 10.1038/s41421-018-0024-3 (PMC5972149; doi:10.1038/s41421-018-0024-3)
Supplement: Supplementary file 1 — Supplementary Figures(PDF 1571 kb) [file 41421_2018_24_MOESM1_ESM.pdf]

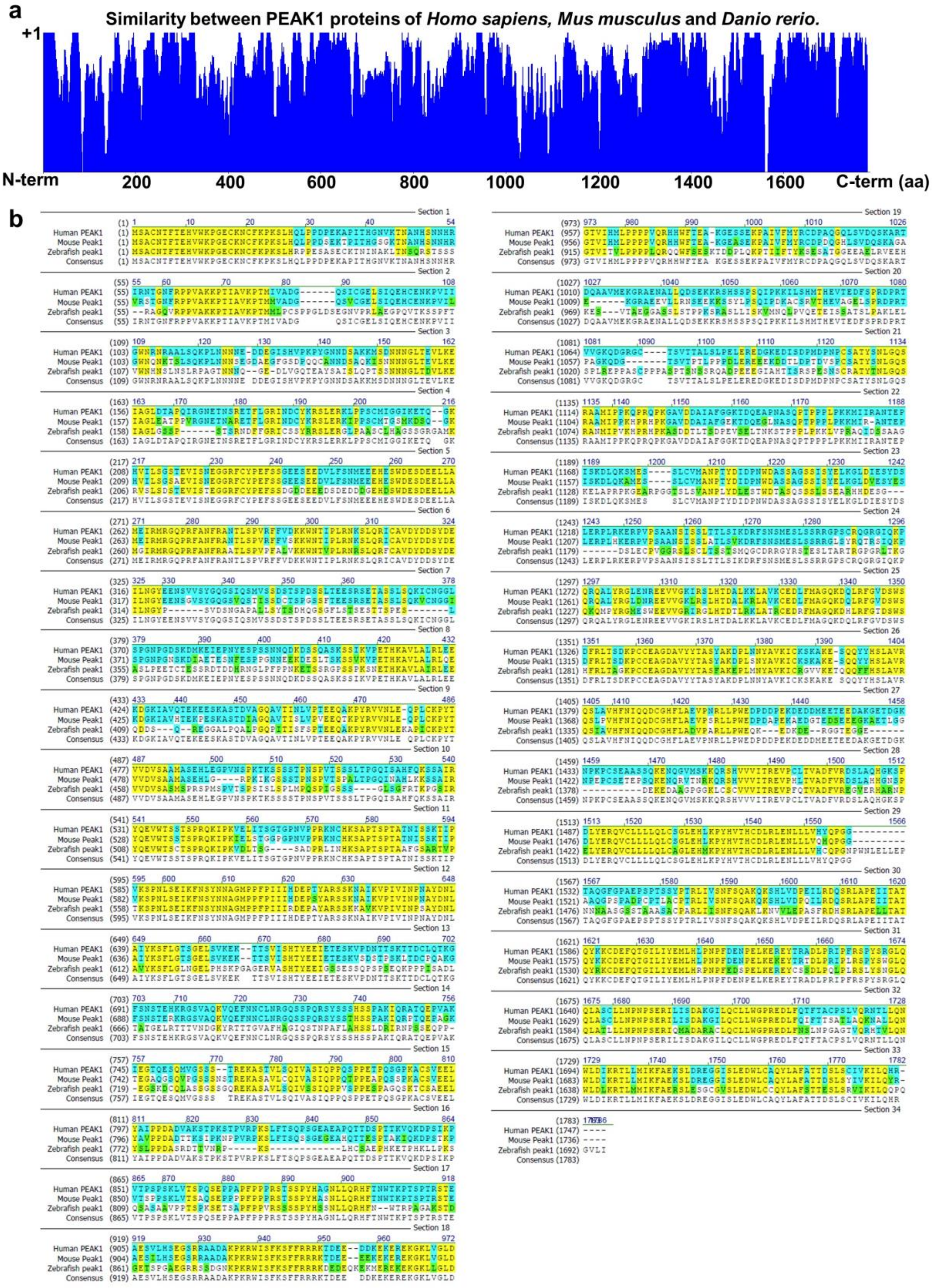
a
Similarity between PEAK1 proteins of Homo sapiens, Mus musculus and Danio rerio.
+1
N-term
200
400
600
800
1000
1200
1400
1600
C-term (aa)
b
Human PEAK1
Mouse Peak1
Zebrafish peak1
Consensus

**Supplementary FigS1**. *peak1* structure shows high conservation across species. (**a**) Comparison of amino acid homology of PEAK1 in human, mouse, and zebrafish. The percentage of identity amino acids of PEAK1 proteins in all species is 44.5%. The percentage of consensus amino acids is 92.0%. (**b**) Complete amino acid sequence homologies shown for human, mouse and zebrafish PEAK1. Yellow back= identical sequences. Blue back= identical sequences in two species. Green back= amino acid consensus sequence.

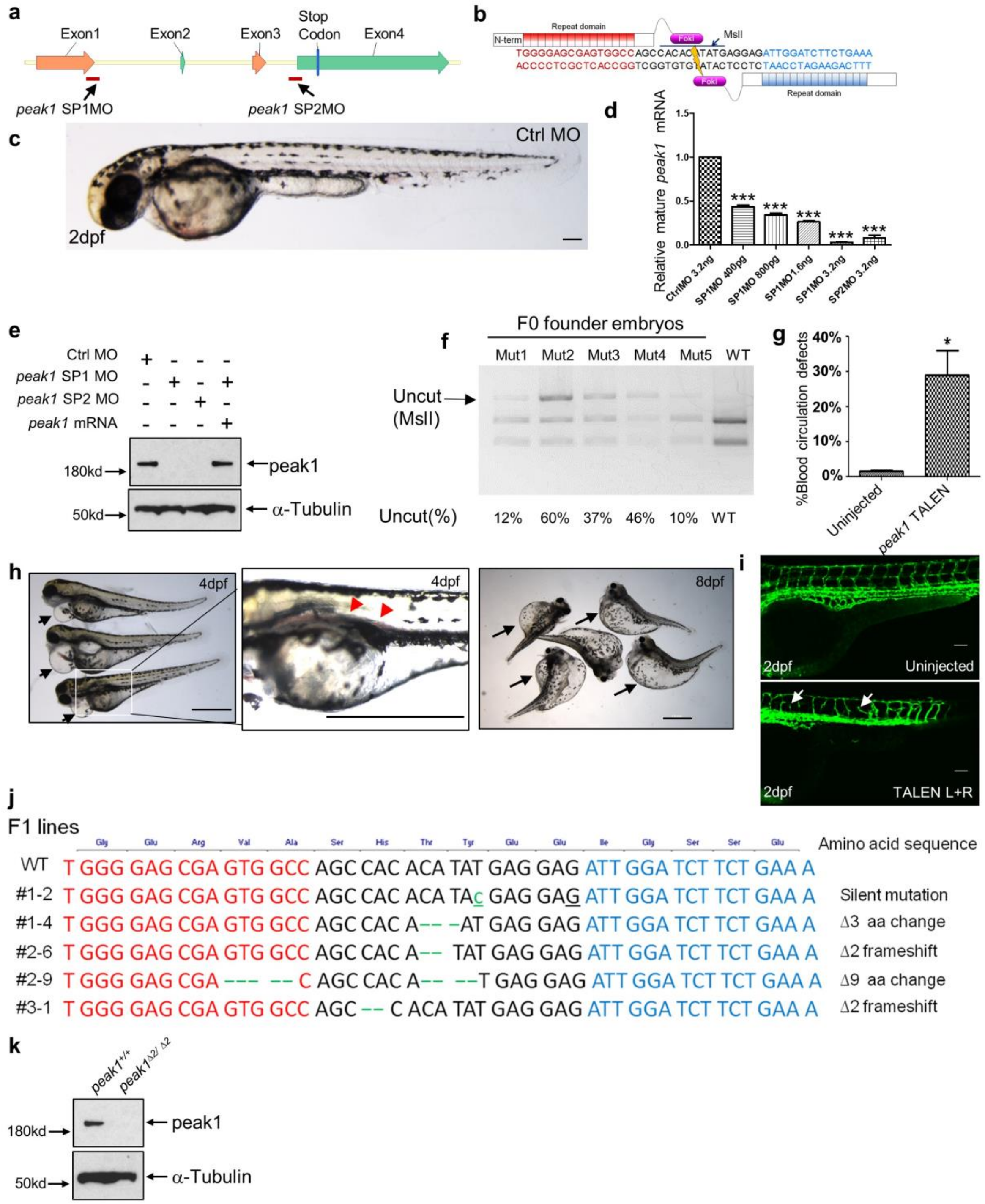


**Supplementary FigS2**. *peak1* is required for vascular development in zebrafish embryos. (**a**) Schematic showing design of splicing morpholino to target the zebrafish *peak1* gene. (**b**) Schematic showing the design of TALEN used to target the zebrafish *peak1* gene. (**c**) Bright field image show normal pericardial cavity and circulation of Ctrl MO injected zebrafish embryos. Scale bar = 50 μm. (**d**) Bar graph represents relative *peak1* mRNA level versus *actn1* from 30 hpf

embryos treated with Ctrl MO, SP1MO or SP2MO and analyzed by qPCR. (**e**) Western blot analyses for indicated proteins of 30 hpf embryos treated as in Figure 1c. (**f**) Genotyping of zebrafish *peak1* TALEN F0 founder embryos. Primers TALEN test F+R and restriction enzyme MslI were used. Uncut (%), the percentage of uncut band out of the total DNA. (**g**) Bar graph represents percentage of animals with blood circulation defects caused by injection of zebrafish *peak1* TALEN mRNAs. (**h**) Bright field images of zebrafish embryos showing severe pericardial edema (arrows) and blood circulation defects (arrowheads) caused by *peak1* TALEN mRNAs at indicated dpf. Scale bar = 1 mm. (**i**) Confocal fluorescence images of the tail vasculature of $Tg(fli1:egfp)^{y1}$ embryos treated with *peak1* TALEN mRNAs were captured at 2dpf. Arrows point to mosaic stunted and disrupted ISV vessels. Scale bar = 50 μm. (**j**) DNA sequencing results of F1 TALEN mutant lines. (**k**) $peak1^{\Delta2/\Delta2}$ TALEN homozygous mutants were incrossed and progeny embryos were lysed at 2dpf and analyzed by western blotting with indicated antibodies. All data are representative of at least three independent experiments. ***, P<0.001; *, P<0.05; N.S., not significant.

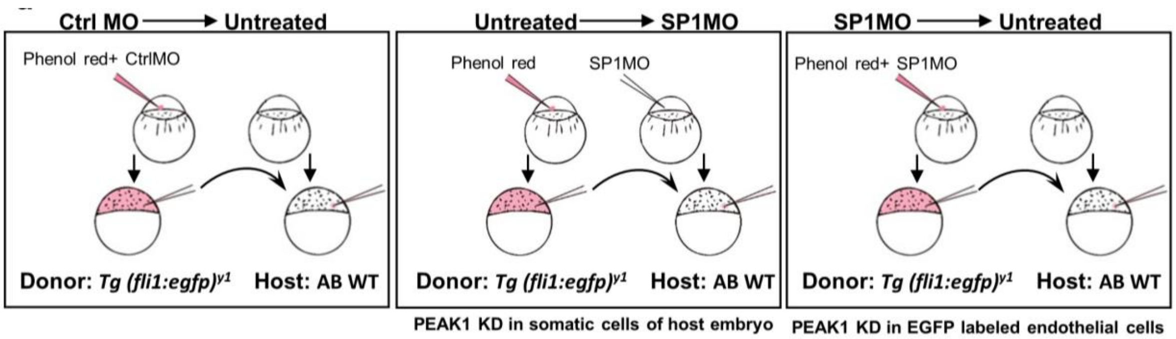


**Supplementary FigS3**. Schematic illustration showing transplantation of endothelial precursor cells from donor *Tg(fli1:egfp)*$^{y1}$ into AB host embryos at the sphere stage. Donor or host embryos were treated with indicated MOs at the one-cell stage.

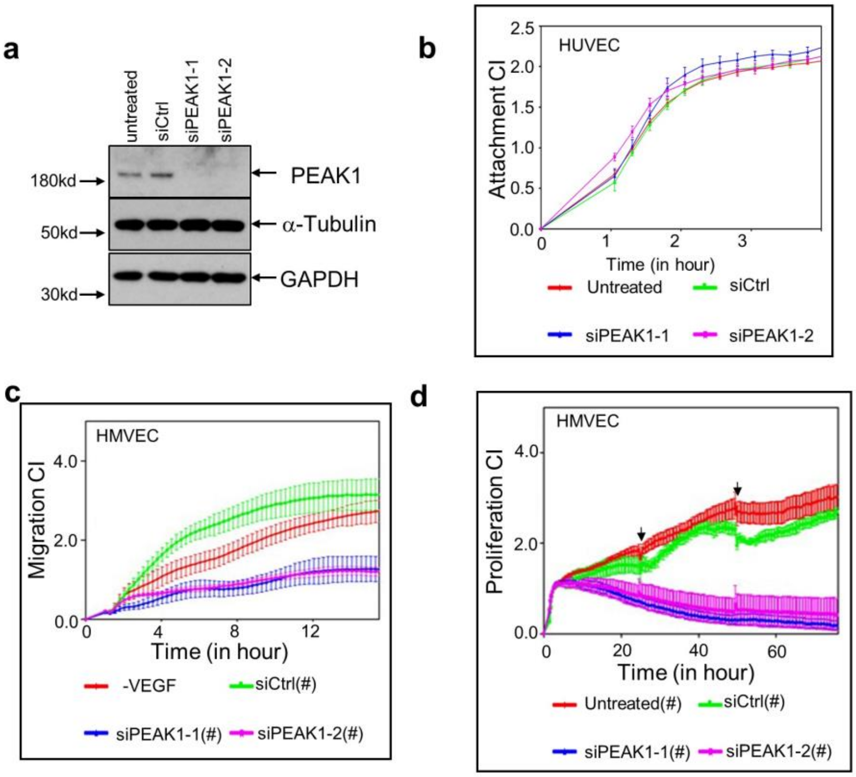


**Supplementary FigS4**. PEAK1 is required for VEGF induced proliferation, migration and morphogenesis of human ECs *in vitro*. (**a**) HUVECs were treated with either siCtrl, siPEAK1-1, or siPEAK1-2. Cells were then lysed and western blotted for the indicated proteins. (**b**) Real time cell attachment kinetics of HUVECs using the xCelligence E16 plate system. Cells were treated as in Figure3E. (**c**) Real-time migration kinetics of HMVECs treated with either siCtrl or two different siRNAs to PEAK1 (siPEAK1-1, siPEAK1-2) in the absence (-VEGF group) or presence of VEGF (# labeled); CI = Cell Index; Mean ± SEM of quadruplicate wells. (**d**) Real-time proliferation kinetics of HMVECs treated as in (**c**) in the presence VEGF. Arrows indicate the time point of adding supplemental VEGF. Mean ± SEM of quadruplicate wells.

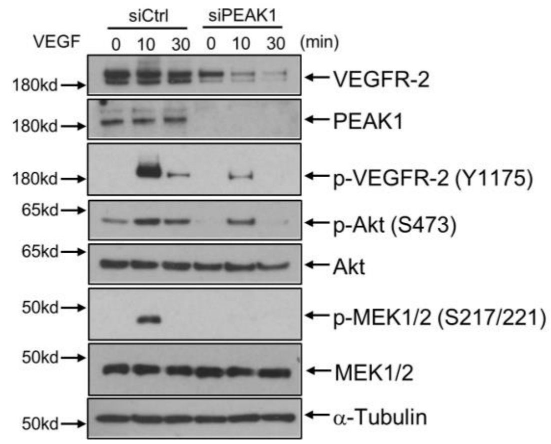


**Supplementary FigS5**. Western blot analysis of HMVECs treated with siRNAs with indicated antibodies after 12 hours of starvation with or without VEGF stimulation.

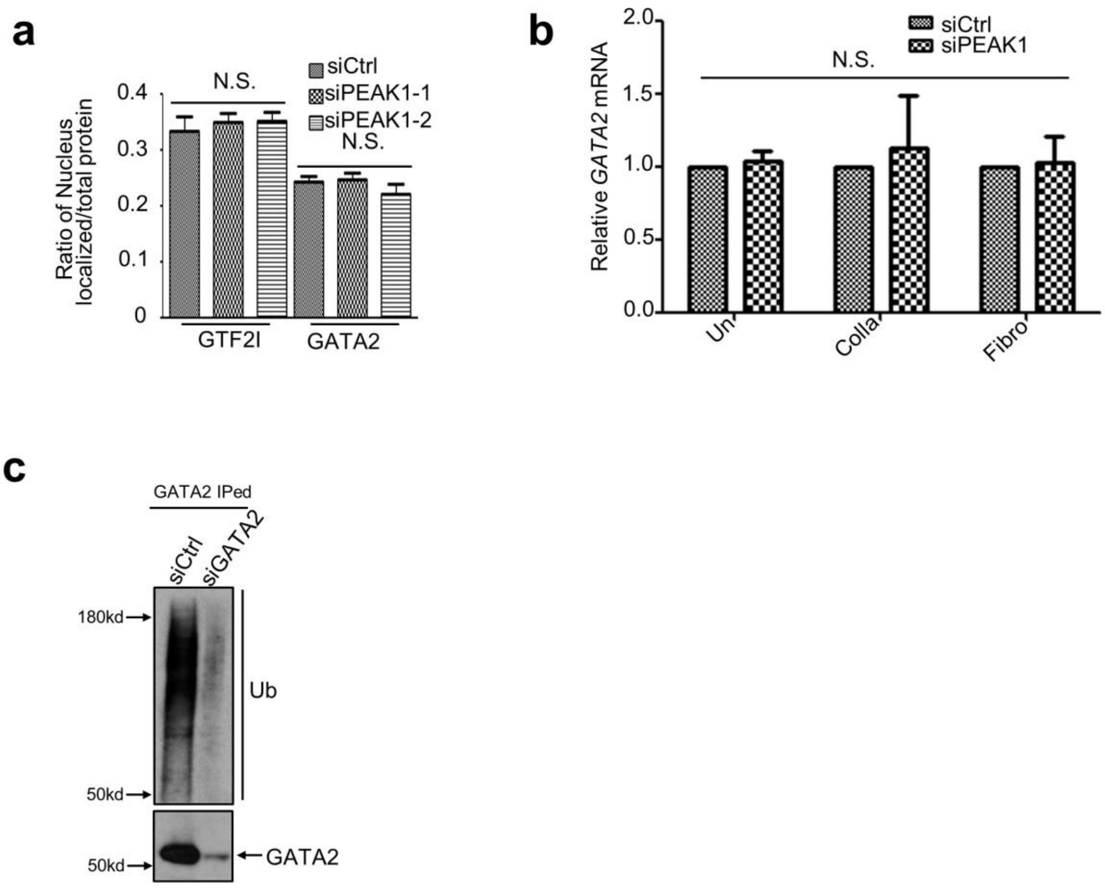


**Supplementary FigS6.** *PEAK1* regulates *VEGFR2* transcription through *GATA2*. (**a**) Bar graph shows the Manders′ coefficient value of GTF2I or GATA2 fluorescent signal versus DAPI nuclear stain of HUVECs cultured on collagen I coated plates and treated with siRNAs. Mean ± SEM; n=10. (**b**) Bar graph represents relative *GATA2* mRNA expression level versus *HPRT1* in HUVECs treated as in Figure7C; Un, uncoated plate; Colla, collagen coated; Fibro, fibronectin coated. (**c**) HUVECs were treated as in Figure 7H. Ubiquitination of IPed GATA2 protein was analyzed by WB. All data are representative of at least three independent experiments. N.S., not significant.

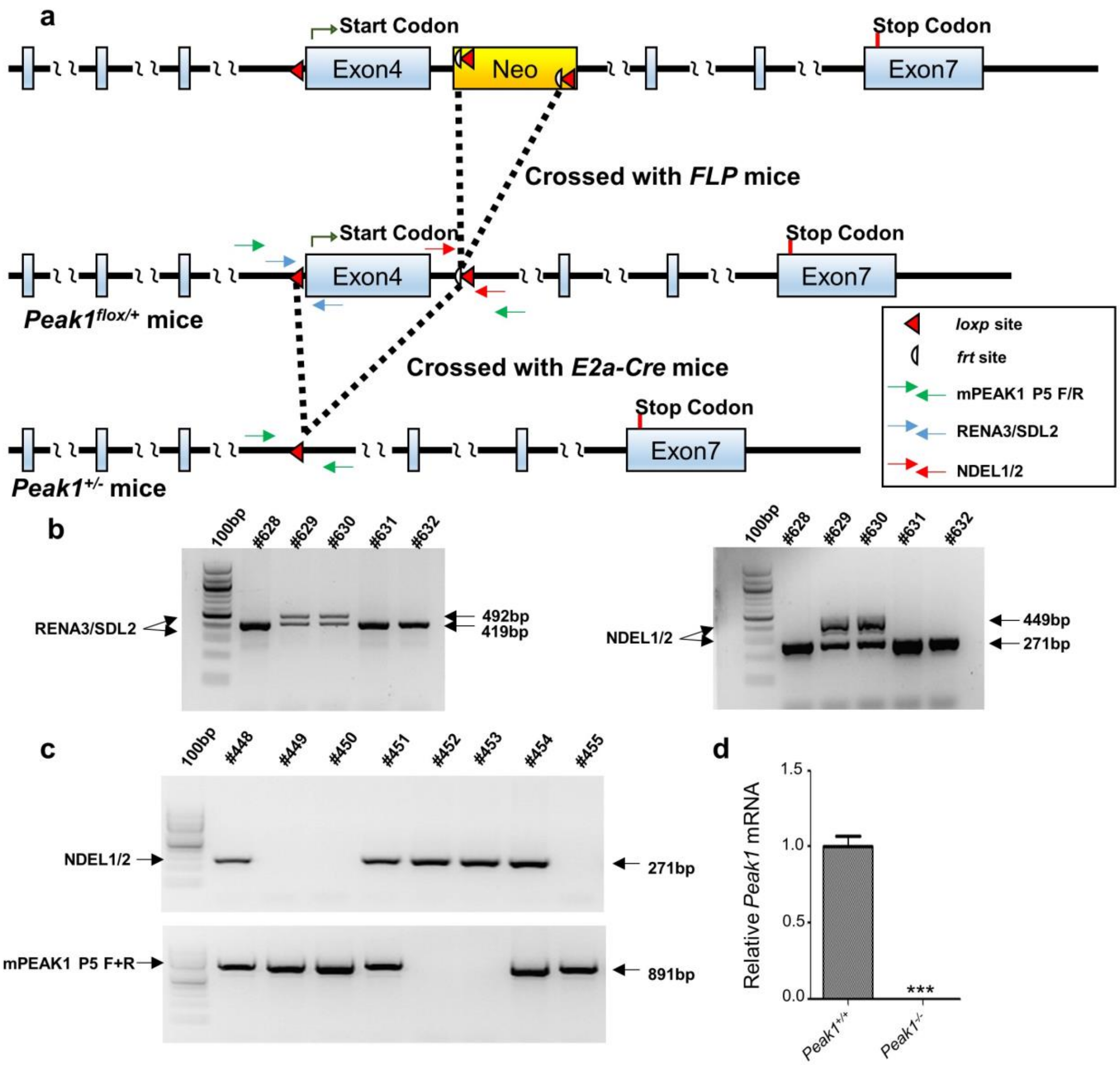


**Supplementary FigS7.** Generation of *Peak1*$^{flox/flox}$ mice and *Peak1*$^{-/-}$ mice. (**a**) Schematic showing the gene targeting strategy to generate *Peak1*$^{flox/+}$mice and *Peak*$^{+/-}$ mice. (**b**) Genotyping of *Peak1*$^{flox/+}$ for the distal loxP insertion with PCR primers RENA3/SDL2, and for the proximal loxP insertion with primers NDEL1/2. (**c**) Genotyping of *Peak1*$^{-/-}$ mice for the genomic deletion of the exon 4 of *Peak1* gene with primers NDEL1/2 and mPEAK1 P5 F+R. (**d**) Bar graph represents relative *Peak1* mRNA level versus *Hprt1* in *Peak1*$^{+/+}$ and *Peak1*$^{-/-}$ mice liver tissue. Mean ± SEM; *n*=3. All data are representative of at least three independent experiments. ***, P<0.001.

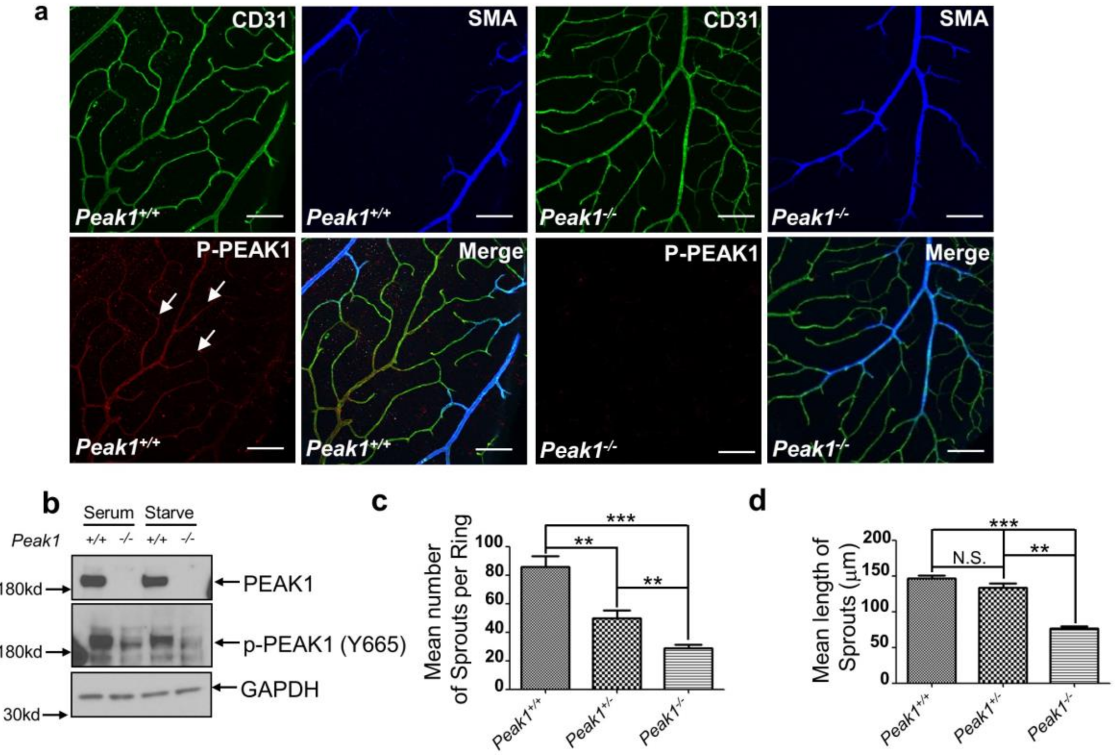


**Supplementary FigS8.** (**a**) Confocal images of *Peak1*$^{-/-}$ and *Peak1*$^{+/+}$ adult mouse retinas stained with the indicated antibodies. CD31 (green). Phosphospecific Y665 PEAK1 (P-PEAK1, red). Smooth muscle actin (SMA, blue). Scale bar = 100 µm. (**b**) Western blot analysis of mouse embryonic fibroblast cells (MEFs) from *Peak1*$^{+/+}$ or *Peak1*$^{-/-}$ mice with the indicated antibodies. (**c**) Bar graph represents the mean number of sprouts per aortic ring from aortic explants treated as in Figure 8D; Mean ± SEM; *n*=10. (**d**) Bar graph represents the average length per sprout from aortic explants treated as in Figure 8d; Mean ± SEM; *n*>=286. ***, P<0.001; (**) P<0.01; *, P<0.05; N.S., not significant.

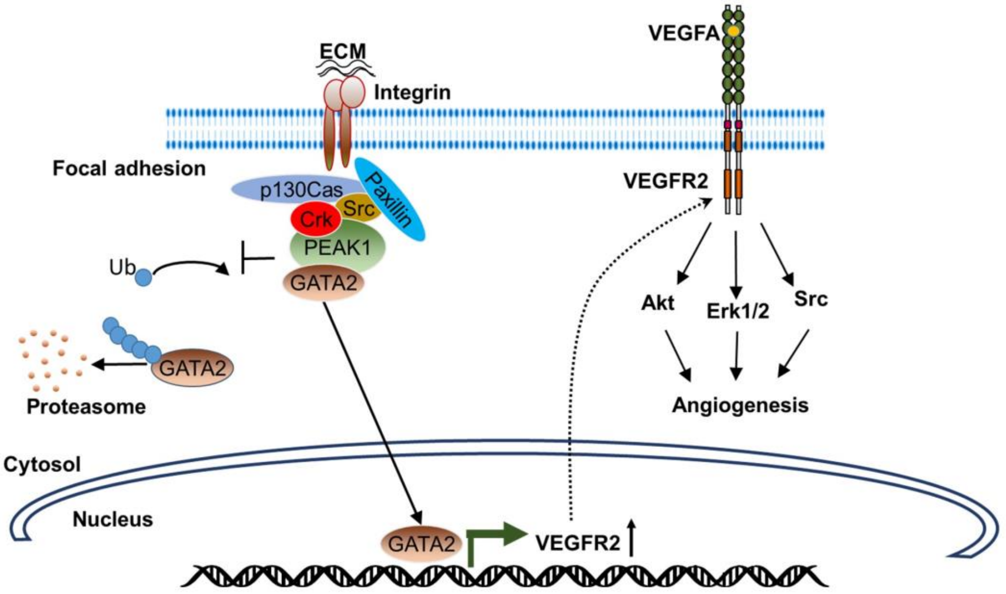


**Supplementary FigS9**. Working model of PEAK1-mediated VEGFR2 expression in ECs. EC attachment to ECM mediates integrin dependent focal adhesion formation and the recruitment of its effector proteins Crk, Src, p130CAS, and paxillin. This complex promotes the interaction of PEAK1 and a transcriptional factor GATA2, and increases GATA2 protein stability by inhibiting GATA2 ubiquitination and destruction by the proteasome. The increased total GATA2 protein levels drives increased VEGFR2 gene transcription as well as increased VEGFR2 protein expression and enhanced EC functions critical for angiogenesis.

**Supplementary Movie S1.** One-cell stage *Tg(fli:nls-egfp)* embryos were injected with Ctrl MO. Time lapse images of ISV ECs expressing nuclear GFP were taken with confocal microscope from 24hpf at 5-minutes intervals.

**Supplementary Movie S2.** One-cell stage *Tg(fli:nls-egfp)* embryos were injected with SP1MO to knockdown *peak1*. Time lapse images of ISV ECs expressing nuclear GFP were taken with confocal microscope from 24hpf at 5-minutes intervals.
