# Supplementary material for: Pseudopodium-enriched atypical kinase 1 mediates angiogenesis by modulating GATA2-dependent VEGFR2 transcription
Source: Cell Discov. 2018 May 29;4:26. doi: 10.1038/s41421-018-0024-3 (PMC5972149; doi:10.1038/s41421-018-0024-3)
Supplement: Supplementary file 2 — Supplementary Tables(DOCX 263 kb) [file 41421_2018_24_MOESM2_ESM.docx]

**Supplementary Table S1**

| Name | Sequence 5'-3' | Usage |
| --- | --- | --- |
| TALEN Test F | GCATCTGGTGGCAGTGAGTA | TALEN mutants genotyping |
| TALEN Test R | CTCGCACAGTGCCAACTAAA |  |
| hPEAK1 F | ACAGGCTTCCAAAAGCTCAA | human *PEAK1* RT-PCR |
| hPEAK1 R | ACAATGGCTGTTCCAGGTTC |  |
| SP1MO test F | TGTCACAGCAGAAGGTGGAG | zebrafish *peak1* SP1MO efficiency test (RT-PCR) |
| SP1MO test R | GAGAGCGTGAGATGGTGTGA |  |
| SP2MO test F | CCGTAAGTTGGCAACCAGAT | zebrafish peak1 SP2MO efficiency test (RT-PCR) |
| SP2MO test R | AGAGTTTCCCACCTGGACCT |  |
| actb1 F | TGGATCAGCAAGCAGGAGTACG | Zebrafish RT-PCR internal control |
| actb1 R | AGGAGGGCAAAGTGGTAAACGC |  |
| zPEAK1 F | ATT GGATCC AGATGGAGGGCTTTCCTGTC | Cloning zebrafish *peak1* CDS into pCS2+ |
| zPEAK1 R | ATT TCTAGA AACGCAAACACATTCACCAA |  |
| mPEAK1 P5 F | TGCCCAAGAATACCTGGTTC | Mouse *Peak1* KO genotyping |
| mPEAK1 P5 R | AGCGCATTCATTTTGCTTCT |  |
| Ndel 1 | CCGTTTGTAGGAAGTGCTTCGTGG |  |
| Ndel 2 | CTTGAAGTTTCATTTCATGTGCATGAGGG |  |
| RENA3 | GAGAGTGGAGCTCAGTGTATCTGAG |  |
| SDL2 | GTCATAGCAGCTCCTCTAAGTACG |  |
| mPEAK1 RT F | CAGACAAAGCCCTACCGTGT | Real time RT-PCR of mouse genes |
| mPEAK1 RT R | GGAACTCCGGGCATAGGATG |  |
| mHprt1 F | GAGGAGTCCTGTTGATGTTGCCAG |  |
| mHprt1 R | GGCTGGCCTATAGGCTCATAGTGC |  |
| mVEGFR2F | TCCAGAATCCTCTTCCATGC |  |
| mVEGFR2R | AAACCTCCTGCAAGCAAATG |  |
| mVEGFR1F | AAGAGAGTCTGGCCTGCTTG |  |
| mVEGFR1R | CTGCTCGGGTGTCTGCTT |  |
| mVEGFR2F | CGGCTCTTTCGCTTACTGTT | Real time RT-PCR of human genes |
| VEGFR2R | CCTGTATGGAGGAGGAGGAA |  |
| VEGFR1F | TGCCACCTCCATGTTTGATG |  |
| VEGFR1R | CCCCGACTCCTTACTTTTACTG |  |
| FGFR1F | AACCTGCCTTATGTCCAGATC |  |
| FGFR1R | AGAGTCCGATAGAGTTACCCG |  |
| HPRT1F | ACCCTTTCCAAATCCTCAGC |  |
| HPRT1R | GTTATGGCGACCCGCAG |  |
| GATA2F | ACAATTTGCACAACAGGTGC |  |
| GATA2R | CACAAGATGAATGGGCAGAA |  |

**Supplementary Table S1 : Primers used in the current study.**

**Supplementary Table S2**

| Primary antibodies |  |  |  |
| --- | --- | --- | --- |
| Name | Company | Catalog | Usage |
| Anti VEGFR-2 | Cell Signaling Tech | #2479 | WB Fig5,6,7 (1:1000) |
| Anti phospho-VEGFR2 (Tyr1175) | Cell Signaling Tech | #2478 | WB Fig5 (1:1000) |
| Anti VEGFR-1 | Cell Signaling Tech | #2893 | WB Fig5 (1:1000) |
| Anti GAPDH | Abcam | #ab22555 | WB Fig5,6,7 (1:5000) |
| Anti alpha tubulin | Cell Signaling Tech | #3873 | WB Fig5,6,7 (1:2000) |
| Anti MEK1/2 | Cell Signaling Tech | #8727 | WB Fig5,7 (1:1000) |
| Anti phospho MEK1/2 (Ser217/221) | Cell Signaling Tech | #9121 | WB Fig5,7 (1:1000) |
| Anti Erk1/2 | Cell Signaling Tech | #9102 | WB Fig5 (1:2000) |
| Anti phospho Erk1/2 (Thr202/Tyr204) | Cell Signaling Tech | #9101 | WB Fig5 (1:5000) |
| Anti Akt | Cell Signaling Tech | #9272 | WB Fig5 (1:1000) |
| Anti phospho Akt (Ser497) | Cell Signaling Tech | #4060 | WB Fig5,7 (1:1000) |
| Anti Src | Cell Signaling Tech | #2109 | WB Fig5 (1:1000) |
| Anti phospho Src family (Tyr 416) | Cell Signaling Tech | #2101 | WB Fig5 (1:1000) |
| Anti GATA2 | Abcam | #ab109241 | WB Fig6,7 (1:1000) |
| Anti GATA2 | Santa Cruz | #SC-9008 | IF Fig6 (1:100) |
| mouse anti-GATA2 AC | Santa-Cruz | #SC-267 AC | IP Fig7 |
| normal mouse IgG-AC | Santa-Cruz | #SC-2343 | IP Fig7 |
| Anti TFII I (GTF2I) | BD | #610942 | WB Fig7 (1:1000); IF Fig6 (1:100) |
| Anti RhoGAP P190 | BD | #610149 | WB Fig6 (1:1000) |
| Anti Ubiquitin antibody | Cell Signaling Tech | #3933 | WB Fig7 |
| Anti PEAK1 (human) | EMD Millipore | #09-274 | WB Fig5,6,7 (1:500); IF Fig 8 (1:100) |
| Anti PEAK1 (mouse) | AVIVA systems | #OAAB11213 | WB Online Fig.V (1:500) |
| Anti PEAK1 (zebrafish) | LSBio | #AP32621PU-N | WB Online Fig.II (1:500) |
| Anti phospho PEAK1 (Tyr665) | EMD Millipore | #ABT52 | IF Fig2 and Online Fig.IV (1:100) |
| Anti CD31 | BD | #550274 | IF Fig2 and Online Fig.IV (1:200) |
| Mouse anti-SMA-Cy3 | Sigma Aldrich | #C6198-2ml | IF Fig2 and Online Fig.IV (1:500) |
| Anti CD31 | Abcam | #ab28364 | IF Fig8 (1:100) |
| Secondary antibodies |  |  |  |
| Alexa Fluor 488 Goat anti rat IgG | Life technology | A11006 | IF Fig2 and Online Fig.IV, VIII (1:500) |
| Alexa Fluor 488 Goat anti rabbit IgG | Life technology | A11008 | IF Fig8 and Online Fig.IV, VIII (1:500) |
| Alexa Fluor 568 Goat anti rabbit IgG | Life technology | A11011 | IF Fig3,8 (1:500) |
| HRP- Goat anti Rabbit IgG | Jackson ImmunoRe | 111-035-003 | WB Fig 6,7,8 (1:10000) |
| HRP- Goat anti mouse IgG | Jackson ImmunoRe | 115-036-068 | WB Fig 6,7,8 (1:10000) |

**Supplementary Table S2: Antibodies used in the current study.**

**Supplementary Table S3**

| Name | Company | Catalog | Targeting region |
| --- | --- | --- | --- |
| siPEAK1-1 | Sigma Aldrich | SASI_Hs02_00357289 | Coding region |
| siPEAK1-2 | Sigma Aldrich | SASI_Hs01_00249365 | Coding region |
| siPEAK1-3 | Qiagen | Hs_KIAA2002_5 | 3'UTR |
| siGATA2-1 | Sigma Aldrich | SASI_Hs01_00106113 | 3'UTR |
| siGATA2-2 | Sigma Aldrich | SASI_Hs01_00106115 | Coding region |
| siCtrl | Sigma Aldrich | SIC001 | Universal control |

**Supplementary Table S3: siRNAs used in the current study.**

**Supplementary Table S6**

**
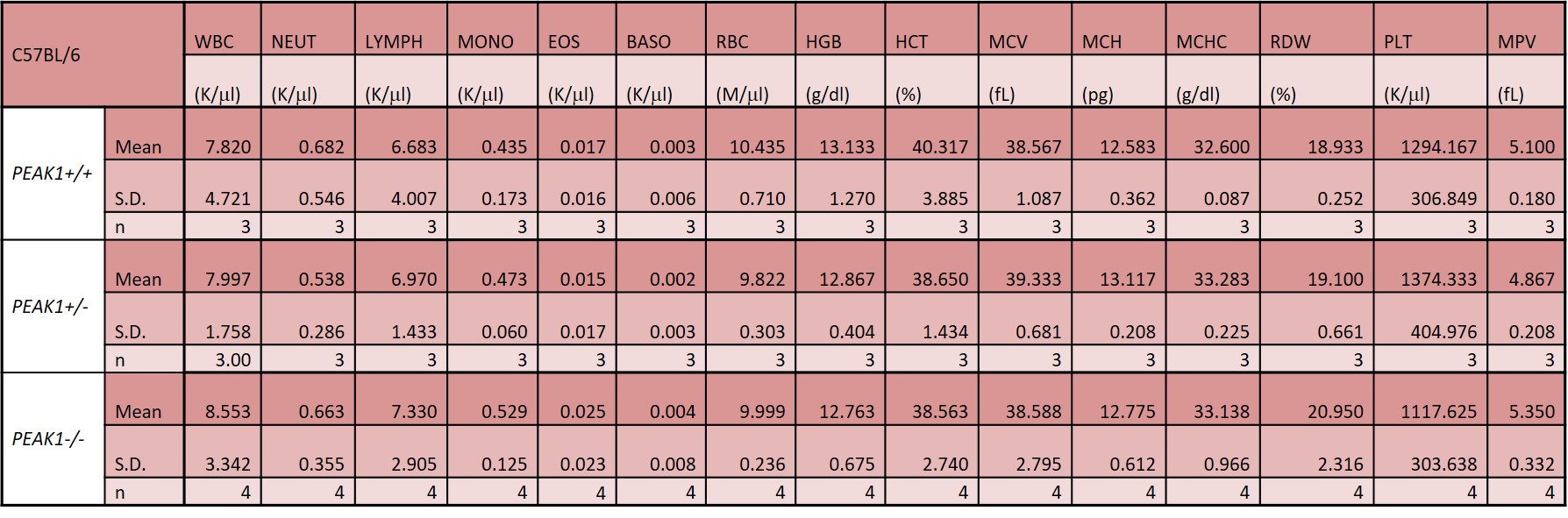
**

**Supplementary Table S6: Complete blood count in *peak1* wildtype, heterozygous, and homozygous knockout mice.**

**Supplementary Table S7**

| **Cancer type** | ***GATA2_VEGFR2*** | ***PEAK1_VEGFR2*** | ***PEAK1_GATA2*** |
| --- | --- | --- | --- |
| ACC 79 | 0.22 | 0.19 | 0.28 |
| BLCA 408 | 0.2 | 0.5 | -0.04 |
| BRCA 1100 | 0.2 | 0.64 | 0.07 |
| CESC 306 | 0.16 | 0.48 | 0.11 |
| CHOL 36 | 0.35 | 0.52 | 0.38 |
| COAD 287 | 0.29 | 0.63 | 0.41 |
| DLBC 28 | 0.59 | 0.73 | 0.48 |
| ESCA 185 | 0.28 | 0.55 | 0.27 |
| GBM 166 | 0.3 | 0.3 | 0.06 |
| HNSC 522 | 0.26 | 0.74 | 0.2 |
| KICH 66 | 0.22 | 0.4 | -0.05 |
| KIRC 534 | 0.51 | 0.62 | 0.35 |
| KIRP 291 | 0.36 | 0.25 | 0.29 |
| LAML 173 | -0.11 | 0.16 | 0.12 |
| LGG 530 | 0.1 | 0.2 | 0.04 |
| LIHC 373 | 0.48 | 0.52 | 0.33 |
| LUAD 517 | 0.23 | 0.38 | 0.31 |
| LUSC 501 | 0.18 | 0.54 | 0.14 |
| MESO 86 | -0.26 | 0.24 | 0.3 |
| OV 307 | 0.21 | 0.39 | 0.09 |
| PAAD 179 | 0.43 | 0.55 | 0.18 |
| PCPG 184 | 0.07 | 0.63 | -0.06 |
| PRAD 498 | -0.16 | 0.75 | -0.14 |
| READ 95 | 0.27 | 0.66 | 0.32 |
| SARC 262 | 0.25 | 0.55 | 0.22 |
| SKCM 471 | 0.31 | 0.3 | 0.01 |
| TGCT 156 | 0.72 | 0.36 | 0.36 |
| THCA 509 | 0.76 | 0.22 | 0.2 |
| THYM 120 | 0.67 | 0.82 | 0.6 |
| UCEC 177 | 0.23 | 0.6 | 0.03 |
| UCS 57 | 0.47 | 0.45 | 0.16 |
| UVM 80 | 0.38 | 0.41 | -0.05 |

**Supplementary Table S7: Correlation of *PEAK1*, *VEGFR2* and *GATA2* mRNA levels in different cancer types.** The Pearson's correlation coefficient between *GATA2* and *VEGFR2*, *PEAK1* and *VEGFR2*, or *PEAK1* and *GATA2* mRNA expression in each cancer type is shown. The value > 0.50 is considered significantly correlated and is highlighted. ACC, Adrenocortical carcinoma; BLCA, Bladder Urothelial Carcinoma; BRCA, Breast invasive carcinoma; CESC, Cervical squamous cell carcinoma and endocervical adenocarcinoma; CHOL, Cholangiocarcinoma; COAD, Colon adenocarcinoma; DLBC, Lymphoid Neoplasm Diffuse Large B-cell Lymphoma; ESCA, Esophageal carcinoma; GBM, Glioblastoma multiforme; HNSC, Head and Neck squamous cell carcinoma; KICH, Kidney Chromophobe; KIRC, Kidney renal clear cell carcinoma; KIRP, Kidney renal papillary cell carcinoma; LAML, Acute Myeloid Leukemia; LGG, Brain Lower Grade Glioma; LIHC, Liver hepatocellular carcinoma; LUAD, Lung adenocarcinoma; LUSC, Lung squamous cell carcinoma; MESO, Mesothelioma; OV, Ovarian serous cystadenocarcinoma; PAAD, Pancreatic adenocarcinoma; PCPG, Pheochromocytoma and Paraganglioma; PRAD, Prostate adenocarcinoma; READ, Rectum adenocarcinoma; SARC, Sarcoma; SKCM, Skin Cutaneous Melanoma; TGCT, Testicular Germ Cell Tumors; THCA, Thyroid carcinoma; THYM, Thymoma; UCEC, Uterine Corpus Endometrial Carcinoma; UCS, Uterine Carcinosarcoma; UVM, Uveal Melanoma.
