# Supplementary material for: Pseudopodium-enriched atypical kinase 1 mediates angiogenesis by modulating GATA2-dependent VEGFR2 transcription
Source: Cell Discov. 2018 May 29;4:26. doi: 10.1038/s41421-018-0024-3 (PMC5972149; doi:10.1038/s41421-018-0024-3)
Supplement: Supplementary file 4 — Supplementary Table S5(DOCX 23 kb) [file 41421_2018_24_MOESM4_ESM.docx]

**Supplementary Table S5**

| Protein ID | Protein Name | log2 siPEAK1/siCtrl | (-) log10 p-value |
| --- | --- | --- | --- |
| Q9H792 | PEAK1_HUMAN | -3.339047062 | 1.696068769 |
| Q9H9C1 | SPE39_HUMAN | -1.694427192 | 1.582564436 |
| Q12891 | HYAL2_HUMAN | -1.564903359 | 1.310593138 |
| P01130 | LDLR_HUMAN | -1.537296164 | 3.995545004 |
| Q96S16 | JMJD8_HUMAN | -1.427653527 | 1.838614528 |
| P40189 | IL6RB_HUMAN | -1.394539568 | 2.454639217 |
| P35968 | VGFR2_HUMAN | -1.346361804 | 2.592552412 |
| Q9H8G1 | ZN430_HUMAN | -1.342746463 | 1.314511295 |
| Q8IVD9 | NUDC3_HUMAN | -1.27245872 | 1.839023857 |
| P05362 | ICAM1_HUMAN | -1.263344041 | 2.229161532 |
| O75173 | ATS4_HUMAN | -1.17931739 | 2.189301943 |
| Q86T13 | CLC14_HUMAN | -1.176305244 | 1.998719185 |
| Q15758 | AAAT_HUMAN | -1.137158559 | 1.342279694 |
| O15230 | LAMA5_HUMAN | -1.137044444 | 1.302184793 |
| Q8IY47 | KBTB2_HUMAN | -1.102281075 | 2.094607977 |
| O43155 | FLRT2_HUMAN | -1.101706148 | 1.407141174 |
| P42574 | CASP3_HUMAN | -1.080068735 | 1.57221115 |
| Q9UPN4 | CP131_HUMAN | -1.048181426 | 1.509645312 |
| P15291 | B4GT1_HUMAN | -1.041176137 | 1.412430718 |
| Q15437 | SC23B_HUMAN | -1.037468772 | 1.314779068 |
| P21810 | PGS1_HUMAN | -1.026650898 | 1.46034115 |
| P02790 | HEMO_HUMAN | -1.025083755 | 2.303790833 |
| P78504 | JAG1_HUMAN | -1.012585929 | 1.32787571 |
| Q86WA9 | S2611_HUMAN | -0.949632633 | 1.478877687 |
| Q13144 | EI2BE_HUMAN | -0.926481512 | 1.321751278 |
| P37023 | ACVL1_HUMAN | -0.896217596 | 1.49515394 |
| Q68D91 | MBLC2_HUMAN | -0.885377937 | 1.373508674 |
| Q9Y3S2 | ZN330_HUMAN | -0.875678408 | 3.27310262 |
| O75191 | XYLB_HUMAN | -0.851329641 | 1.751817977 |
| Q8IWA5 | CTL2_HUMAN | -0.822779403 | 1.647595155 |
| P23470 | PTPRG_HUMAN | -0.799175102 | 1.81317105 |
| Q9HBU6 | EKI1_HUMAN | -0.786382684 | 1.519536762 |
| O95864 | FADS2_HUMAN | -0.781975508 | 2.574931415 |
| P03956 | MMP1_HUMAN | -0.771666734 | 2.662909599 |
| O15127 | SCAM2_HUMAN | -0.76940956 | 1.373930881 |
| Q9NV88 | INT9_HUMAN | -0.751898869 | 1.701275472 |
| O95155 | UBE4B_HUMAN | -0.742270576 | 1.335755079 |
| Q9ULV1 | FZD4_HUMAN | -0.738457494 | 2.139464508 |
| Q9BV57 | MTND_HUMAN | -0.737581797 | 1.488886648 |
| Q96G01 | BICD1_HUMAN | -0.736907169 | 1.996118797 |
| Q53EP0 | FND3B_HUMAN | -0.721776197 | 3.3953502 |
| P56937 | DHB7_HUMAN | -0.71219976 | 1.791587084 |
| Q92823 | NRCAM_HUMAN | -0.694716454 | 2.98177592 |
| Q8N766 | EMC1_HUMAN | -0.67677144 | 1.763841836 |
| Q6P3W7 | SCYL2_HUMAN | -0.672042773 | 1.846690339 |
| Q9P206 | K1522_HUMAN | -0.657536259 | 1.321557583 |
| Q13772 | NCOA4_HUMAN | -0.652396104 | 1.600377976 |
| Q9BSB4 | ATGA1_HUMAN | -0.642744448 | 1.358054638 |
| Q9C0D5 | TANC1_HUMAN | -0.640552858 | 1.425523994 |
| Q04721 | NOTC2_HUMAN | -0.637072627 | 2.141262199 |
| Q9NR09 | BIRC6_HUMAN | -0.635135349 | 1.59228872 |
| P46531 | NOTC1_HUMAN | -0.630045177 | 1.886271157 |
| Q9H0M0 | WWP1_HUMAN | -0.619783191 | 1.46137621 |
| Q66K14 | TBC9B_HUMAN | -0.616974627 | 1.351464811 |
| Q86U38 | NOP9_HUMAN | -0.615688278 | 2.845849382 |
| P11717 | MPRI_HUMAN | -0.609459553 | 1.965043534 |
| O00501 | CLD5_HUMAN | -0.606399232 | 1.385035706 |
| P33316 | DUT_HUMAN | -0.5910153 | 1.947265655 |
| Q96P48 | ARAP1_HUMAN | -0.589479582 | 1.490418242 |
| Q92572 | AP3S1_HUMAN | -0.589236368 | 1.609785304 |
| Q9NY35 | CLDN1_HUMAN | -0.583287364 | 1.977052785 |
| Q9H773 | DCTP1_HUMAN | -0.572251504 | 1.327408087 |
| Q9BQS8 | FYCO1_HUMAN | -0.568403384 | 1.410159861 |
| Q460N5 | PAR14_HUMAN | 0.588198579 | 2.349530916 |
| O95232 | LC7L3_HUMAN | 0.59090603 | 1.452190662 |
| P19634 | SL9A1_HUMAN | 0.594330895 | 1.328444926 |
| Q9UH62 | ARMX3_HUMAN | 0.594949285 | 1.746520702 |
| O95749 | GGPPS_HUMAN | 0.595558754 | 1.658070052 |
| P19022 | CADH2_HUMAN | 0.597645578 | 1.994788486 |
| P13797 | PLST_HUMAN | 0.600041132 | 1.55914804 |
| O43592 | XPOT_HUMAN | 0.600774347 | 1.85120379 |
| Q96DH6 | MSI2H_HUMAN | 0.603368284 | 1.452349644 |
| Q9NP79 | VTA1_HUMAN | 0.606618056 | 3.562418903 |
| P08253 | MMP2_HUMAN | 0.606958594 | 1.347514918 |
| P55263 | ADK_HUMAN | 0.608883542 | 1.783773153 |
| Q12765 | SCRN1_HUMAN | 0.609231995 | 1.30902732 |
| Q96FV9 | THOC1_HUMAN | 0.610066267 | 1.373371717 |
| P63151 | 2ABA_HUMAN | 0.610630036 | 1.450934256 |
| P62380 | TBPL1_HUMAN | 0.610937454 | 1.613039666 |
| O95786 | DDX58_HUMAN | 0.615262261 | 1.846442067 |
| Q6N069 | NAA16_HUMAN | 0.616756833 | 1.663696013 |
| Q6NYC1 | JMJD6_HUMAN | 0.621485803 | 1.828974834 |
| Q86UA1 | PRP39_HUMAN | 0.621823425 | 1.47983968 |
| P12429 | ANXA3_HUMAN | 0.638063127 | 1.395504902 |
| Q8IVH8 | M4K3_HUMAN | 0.638475424 | 1.408720857 |
| Q9NVG8 | TBC13_HUMAN | 0.651820942 | 1.350888175 |
| O43148 | MCES_HUMAN | 0.652917545 | 1.898656883 |
| O75663 | TIPRL_HUMAN | 0.654030582 | 1.342785595 |
| Q9UBB4 | ATX10_HUMAN | 0.656429334 | 1.99538778 |
| P50895 | BCAM_HUMAN | 0.659506681 | 1.619456588 |
| Q96K12 | FACR2_HUMAN | 0.66047169 | 1.773142606 |
| Q8NDZ4 | DIA1_HUMAN | 0.662045792 | 1.578580695 |
| Q86Y07 | VRK2_HUMAN | 0.67064669 | 2.131250723 |
| Q92466 | DDB2_HUMAN | 0.672052692 | 1.332812266 |
| P49137 | MAPK2_HUMAN | 0.680937641 | 1.791350902 |
| Q96DG6 | CMBL_HUMAN | 0.68237917 | 1.552641379 |
| O14896 | IRF6_HUMAN | 0.684401557 | 1.867113113 |
| O00712 | NFIB_HUMAN | 0.685269518 | 2.065086546 |
| Q9H446 | RWDD1_HUMAN | 0.691812068 | 1.446222621 |
| P32321 | DCTD_HUMAN | 0.692228971 | 1.820448733 |
| Q13242 | SRSF9_HUMAN | 0.701506432 | 1.952578631 |
| P62158 | CALM_HUMAN | 0.702637508 | 1.562797845 |
| Q8WVJ2 | NUDC2_HUMAN | 0.7137853 | 1.854989438 |
| Q4L180 | FIL1L_HUMAN | 0.727325227 | 1.785157939 |
| P23443 | KS6B1_HUMAN | 0.729483202 | 2.033651739 |
| Q9NRG1 | PRDC1_HUMAN | 0.736146898 | 1.658951572 |
| P23528 | COF1_HUMAN | 0.736401161 | 1.348584522 |
| Q96BP3 | PPWD1_HUMAN | 0.739245776 | 2.036660736 |
| Q9P0V3 | SH3B4_HUMAN | 0.754786845 | 1.359601499 |
| Q8IZQ1 | WDFY3_HUMAN | 0.756609234 | 2.147827813 |
| Q15327 | ANKR1_HUMAN | 0.756997939 | 2.732877576 |
| Q7Z6J6 | FRMD5_HUMAN | 0.761971678 | 1.844199038 |
| Q00534 | CDK6_HUMAN | 0.770430329 | 2.566158961 |
| Q9HBF4 | ZFYV1_HUMAN | 0.776234085 | 1.850014036 |
| Q9Y3Z3 | SAMH1_HUMAN | 0.787866049 | 2.087418549 |
| O95999 | BCL10_HUMAN | 0.789847842 | 1.53598024 |
| O14646 | CHD1_HUMAN | 0.792168721 | 2.035445116 |
| O60447 | EVI5_HUMAN | 0.793840815 | 2.225455943 |
| O60879 | DIAP2_HUMAN | 0.795514503 | 1.393598508 |
| P42224 | STAT1_HUMAN | 0.807935755 | 2.526390367 |
| Q9H993 | ARMT1_HUMAN | 0.809665999 | 1.606833135 |
| Q9Y2H1 | ST38L_HUMAN | 0.822437128 | 1.77373 |
| Q8WX93 | PALLD_HUMAN | 0.83789883 | 2.056134542 |
| Q9NRX4 | PHP14_HUMAN | 0.841423432 | 1.961242172 |
| P52566 | GDIR2_HUMAN | 0.844432179 | 1.840865699 |
| O75475 | PSIP1_HUMAN | 0.861707848 | 1.321566603 |
| Q9BSE4 | HERP2_HUMAN | 0.864148657 | 1.395739925 |
| P47974 | TISD_HUMAN | 0.87625701 | 1.963201502 |
| Q8N5U6 | RNF10_HUMAN | 0.901236814 | 2.222828534 |
| Q8N4J0 | CARME_HUMAN | 0.902783197 | 1.656100675 |
| O60583 | CCNT2_HUMAN | 0.916713511 | 1.467275518 |
| P80217 | IN35_HUMAN | 0.939086647 | 1.8182374 |
| P00813 | ADA_HUMAN | 0.940979541 | 1.557150279 |
| Q8IYB3 | SRRM1_HUMAN | 0.957226153 | 1.341311526 |
| Q96BT7 | ALKB8_HUMAN | 0.972217745 | 1.767693851 |
| Q8TDB6 | DTX3L_HUMAN | 0.983925104 | 1.908562014 |
| Q9UII4 | HERC5_HUMAN | 1.025183659 | 1.473781521 |
| Q9H0F7 | ARL6_HUMAN | 1.030889097 | 1.710483704 |
| P09429 | HMGB1_HUMAN | 1.032922877 | 1.439291676 |
| O94760 | DDAH1_HUMAN | 1.03415466 | 1.537153705 |
| O00623 | PEX12_HUMAN | 1.053877966 | 1.502134523 |
| O00330 | ODPX_HUMAN | 1.060136299 | 1.554579038 |
| P45379 | TNNT2_HUMAN | 1.066137369 | 1.356870052 |
| Q8IXQ6 | PARP9_HUMAN | 1.082305048 | 3.174511967 |
| P23497 | SP100_HUMAN | 1.088517008 | 1.530261073 |
| Q9P032 | NDUF4_HUMAN | 1.099955578 | 1.369976415 |
| Q92994 | TF3B_HUMAN | 1.126662863 | 1.404694159 |
| Q13287 | NMI_HUMAN | 1.137046422 | 1.842311506 |
| Q13325 | IFIT5_HUMAN | 1.138570749 | 1.530328348 |
| Q01804 | OTUD4_HUMAN | 1.146026339 | 1.811310635 |
| O00635 | TRI38_HUMAN | 1.164614505 | 2.096878439 |
| Q9UEE5 | ST17A_HUMAN | 1.175754119 | 1.907283241 |
| Q5FYB0 | ARSJ_HUMAN | 1.196319424 | 1.636316713 |
| A4D1E9 | GTPBA_HUMAN | 1.236450688 | 1.615017837 |
| O75508 | CLD11_HUMAN | 1.295265448 | 1.583609721 |
| Q8WXA3 | RUFY2_HUMAN | 1.322415109 | 1.676818298 |
| P19525 | E2AK2_HUMAN | 1.446292116 | 2.338868504 |
| Q9H2H8 | PPIL3_HUMAN | 1.455283549 | 1.310814824 |
| Q5EBM0 | CMPK2_HUMAN | 1.5762599 | 1.901984713 |
| Q8WTS6 | SETD7_HUMAN | 1.604879892 | 2.439800617 |
| P51151 | RAB9A_HUMAN | 1.805629918 | 2.037305177 |
| P00973 | OAS1_HUMAN | 1.927914439 | 3.676533072 |
| Q00978 | IRF9_HUMAN | 1.983702435 | 2.130268288 |
| P09914 | IFIT1_HUMAN | 2.054898074 | 2.735304221 |
| P29728 | OAS2_HUMAN | 2.056533023 | 1.375052265 |
| P20591 | MX1_HUMAN | 2.365291864 | 2.266796782 |

**Table S5: Most up and down regulated proteins in PEAK1 KD HUVECs identified by quantitative multiplexed proteomics.** Top 63 most down-regulated (blue) and 103 most up-regulated (red) proteins (>1.5 fold), reads analyzed from 3 biological replicates using quantitative multiplexed proteomics and sorted by change in expression based on the log ratio between HUVECs treated with siPEAK1 and siCtrl with a P value< 0.05.
